# Salmonella infection and biofilms drive anti-C1q and anti-dsDNA autoantibodies in systemic autoimmunity

**DOI:** 10.1371/journal.ppat.1014666

**Published:** 2026-10-01

**Authors:** Kaitlyn Grando, Molly Elkins, Lauren Nicastro, Shingo Bessho, Sophia Olubajo, Anita Kowal, Vincent Tam, Roberto Caricchio, David Pisetsky, Gregg J. Silverman, Çagla Tükel

**Affiliations:** 1 Center for Microbiology and Immunology, Temple University Lewis Katz School of Medicine, Philadelphia, Pennsylvania, United States of America; 2 Division of Rheumatology, Department of Medicine, UMass Chan Medical School, Worcester, Massachusetts, United States of America; 3 Durham VA Medical Center and Duke University Medical Center, Durham, North Carolina, United States of America; 4 Department of Medicine, NYU Grossman School of Medicine, New York, New York, United States of America; Deutsches Elektronen-Synchrotron, GERMANY

## Abstract

Systemic lupus erythematosus (SLE) is characterized by autoantibody production alongside inflammatory manifestations, including nephritis. In SLE, infections are frequent and associated with disease flares, increasing morbidity and mortality. Additionally, dysbiosis, often marked by overgrowth of Pseudomonadota, is a prominent feature of SLE and lupus nephritis. We previously demonstrated that complexes of DNA and curli, an amyloidogenic protein from biofilms of Pseudomonadota, including *Salmonella* Typhimurium and *E. coli*, can induce anti-dsDNA and anti-chromatin autoantibody responses. Here, we assessed the effects of curli administration or *Salmonella* infection on autoimmune manifestations in wild-type and lupus-prone mice. A multiplex autoantibody assay showed increased levels of anti-C1q antibodies in curli-treated C57BL/6 and lupus-prone NZBxW/F1 mice compared to PBS-treated controls. An anti-C1q-specific ELISA confirmed the presence of anti-C1q antibodies in C57BL/6 mice after treatment with curli/DNA, extracted from *E. coli* or *Salmonella* biofilms, or infection with *Salmonella*. Prior to the onset of spontaneous disease, sera from young NZBxW/F1 mice, injected with curli, demonstrated increased anti-dsDNA and anti-C1q antibody responses compared to PBS-treated controls; however, no differences were found in older mice, post-onset, when autoantibody levels were already high. In MRL/lpr mice, curli treatment significantly exacerbated anti-C1q antibody production regardless of age and disease stage, compared to PBS-treated controls. While curli treatment did not significantly affect proteinuria in either model, curli-treatment of MRL/lpr mice significantly increased C1q deposition in glomeruli. This study uncovers a novel mechanism for the induction of anti-C1q autoantibodies and how infection or dysbiosis may exacerbate renal disease in SLE.

## Author summary

Up to half of patients with systemic lupus erythematosus (SLE) develop lupus nephritis, a major cause of morbidity characterized by immune complex deposition and complement activation within the glomeruli. The deposition of immune complexes is thought to contribute to the development of kidney inflammation and injury. Our previous work found a link between anti-DNA antibody production and exposure to the biofilm matrices of bacteria like *Salmonella* and *E. coli*. Such bacterial infections or microbiota species overgrowths are associated with autoimmunity. Here, we found, for the first time, that purified biofilm matrix or *Salmonella* infection triggers the production of antibodies targeting C1q in both healthy and lupus-prone mouse models. Additionally, we showed that biofilm matrix administration increased C1q deposition in the glomeruli of lupus-prone mice, implicating bacterial biofilms in the exacerbation of lupus nephritis. This work highlights the interplay between environmental insults like bacterial infections, age, and the development or exacerbation of autoimmunity, in genetically predisposed individuals.

## Introduction

Systemic lupus erythematosus (SLE) is a systemic autoimmune disease characterized by tissue inflammation and the production of a broad array of autoantibodies, a common feature of many autoimmune and inflammatory diseases. In SLE, antibodies to nuclear molecules (antinuclear antibodies or ANAs) are a prominent and defining serological feature, with antibodies to dsDNA serving as an important marker for both classification and disease activity. The expression of autoantibodies to cytoplasmic, membrane, and soluble proteins, however, also occurs in SLE, pointing toward a more generalized immune disturbance. While the mechanisms of autoantibody production in SLE are not well-defined, infection has long been considered as a possible trigger. Indeed, the transient production of autoantibodies has been detected in healthy individuals after infections with bacteria, viruses, and parasites [1–3]. Of interest, prior to their description in SLE patients, antibodies against DNA were first identified in patients after bacterial infection [4–7].

In patients with SLE, anti-dsDNA antibodies, along with antibodies to other nuclear molecules—including RNA and complexes of proteins with nucleic acids—can appear up to a decade before the onset of disease symptoms [8,9]; these findings suggest a gradual and perhaps multi-step process by which serological abnormalities culminate into clinical manifestations in genetically susceptible individuals. Of responses in SLE, the origin of anti-dsDNA production has been particularly enigmatic, since immunization of animals with DNA fails to induce antibody responses, even with adjuvant [10]. In contrast, immunization with protein nuclear molecules can lead to autoantibody production [11,12]. One possible explanation for the generation of anti-DNA antibodies is the formation of complexes between an immunogenic protein (carrier) and typically non-immunogenic DNA (hapten) which can induce the production of these antibodies [7,13]. In addition to anti-dsDNA antibodies, many other autoantibodies appear commonly in SLE, including those targeting chromatin, ribonucleoprotein, phospholipids, Ro (SS-A), La (SS-B), HMGB1, Smith antigens (Sm), ribosomal protein, and more are being discovered as technology for finding autoantigens evolves [14].

In SLE, a subset of approximately 40% of patients develops lupus nephritis (LN), inflammation and injury of the kidneys which results in loss of kidney function and significantly higher mortality [15–17]. Lupus nephritis occurs in various patterns; in many patients, active nephritis is associated with rising anti-dsDNA levels and decreasing complement levels [18]. Furthermore, antibodies against complement component C1q are associated with proliferative LN, a form of LN that has higher mortality than other subtypes [19–24]. As shown by renal immunohistology, a diagnostic feature of LN is the presence of immune complexes along the glomerular basement membranes [25,26]. These complexes include immunoglobulin (IgG, IgA, and IgM), including anti-nuclear and anti-C1q autoantibodies, and complement components (C1q and C3) in a so-called “full-house” pattern [27–32]. While anti-DNA antibodies may form immune complexes with DNA to deposit in the kidney, the mechanisms by which anti-C1q antibodies promote nephritis remain unclear. Possibilities include C1q depletion leading to impaired clearance of apoptotic bodies, and/or localized complement activation from immune complex deposition, promoting inflammation and tissue damage [32–35].

Infections are a leading cause of morbidity and mortality in SLE [36–38] and are often associated with disease flares [39–41]. *E. coli*, *Staphylococcus,* and *Streptococcus* infections are particularly widespread [42,43]. Non-typhoidal *Salmonella,* which causes gastroenteritis in immunocompetent patients, can cause invasive infections in SLE patients including soft tissue infections and sepsis [44–48]. Additionally, dysbiosis of the gut microbiota has been implicated in the development of autoimmune diseases and, in particular, disease flares in SLE [49–51]. Overgrowth of Pseudomonadota (formerly Proteobacteria) is a signature of dysbiosis, including that occurring in SLE patients, especially those with LN [52–56]. This overgrowth may be driven by the competitive advantage that Pseudomonadota like *E. coli* and *Salmonella* have in the inflammatory environment in the gut [57–62]. Our group demonstrated that complexes of DNA with the protein curli from the biofilms of non-typhoidal *Salmonella enterica* serovar Typhimurium (STm) and *E. coli,* can induce the production of anti-dsDNA and anti-chromatin autoantibodies, both in wild-type and lupus-prone NZBxW/F1 mice [63–66].

Many species of bacteria form biofilms, multicellular communities embedded in an extracellular matrix (ECM). These biofilms represent a unique environment for bacterial growth, providing nutrient support as well as protection from the immune system and agents like antibiotics. The ECM of biofilms are decorated with DNA, proteins, and carbohydrates to provide barrier function, structural scaffolding, and surface adhesion [67–71]. Curli is an abundant secreted protein that comprises 85% of the ECM of Pseudomonadota biofilms [70,72,73]. Curli forms amyloid-like fibrils which, notably, bind extruded bacterial DNA within the ECM. Such curli/DNA complexes are far more immunogenic than either curli or DNA alone [64,74,75]. Curli-containing biofilms are present in the gastrointestinal tract of infected mice [65,76,77].

Previous studies from our group demonstrated that curli/DNA complexes, when they escape from the gut [65,76], activate multiple innate immune receptors, including TLR2 [78,79], TLR9 [75], NLRP3 inflammasome [80], and the unfolded protein response [76]. The result is the production of pro-inflammatory cytokines, activation of the type I IFN and Th17 responses [63,64,66,81,82], and joint inflammation resembling reactive arthritis [63,65], an autoimmune disease that can occur following infection with invasive bacteria. We also observed that SLE patients had elevated anti-curli antibodies that correlated with disease flares and persistent bacteriuria with curli-producing *E. coli* [41].

In this study, we were interested in determining whether components of biofilms can exacerbate or accelerate SLE in mouse models at various stages of disease, alter disease manifestations (i.e., renal involvement), or induce lupus-associated autoantibodies against additional targets, besides dsDNA or chromatin. As reported herein, we found that administration of extracted biofilm material that contains curli and DNA, or oral infection with STm, resulted in elevated levels of anti-C1q autoantibodies in wild-type and lupus-prone NZBxW/F1 mice. Additionally, we found that, after curli/DNA treatment, lupus-prone MRL/lpr mice displayed a dramatic increase in levels of anti-C1q antibodies and elevated C1q deposition in the glomeruli. These results suggest a novel mechanism that connects infection with organisms producing biofilms, autoantibody production, and development of severe nephritis in SLE.

## Results

### Curli treatment or infection with STm induces anti-C1q autoantibodies

Previously, we demonstrated that intraperitoneal (i.p.) administration of curli/DNA purified from *Salmonella enterica* serovar Typhimurium (STm) biofilms; i.p infection with *E. coli* biofilms which contain curli/DNA; and oral infection with STm which form curli-containing biofilms in the gut in vivo [65,77], can trigger the production of anti-dsDNA and anti-chromatin autoantibodies in wild-type mice [63–66]. We were therefore interested in whether mice develop other autoantibodies characteristic of SLE in response to curli exposure, either in wild-type mice or in the context of genetic predisposition to autoimmune disease. For this purpose, we performed a multiplex bead-based Luminex assay for antibodies against antigens associated with SLE. Serum was collected from wild-type C57BL/6 or lupus-prone NZBxW/F1 mice injected i.p. with purified curli or PBS (control). Importantly, injections were initiated at 6–8 weeks of age, before autoantibody production typically begins in NZBxW/F1 mice. In addition to the expected anti-dsDNA and anti-chromatin responses, one of the strongest responses observed were antibodies directed against complement component C1q; levels of these antibodies appeared elevated in both curli-treated C57BL/6 and NZBxW/F1 mice compared to PBS-injected controls, though only NZBxW/F1 mice reached statistical significance (Fig 1A-B, S1 Fig). Other curli-associated responses of note included low levels of antibodies to ribosomal protein P2 in both C57BL/6 and NZBxW/F1 mice, and HMGB1 and La (SS-B) in NZBxW/F1 mice, which did not reach statistical significance due to a high degree of variability between individuals (S1 Fig). We chose to pursue anti-C1q antibodies further for this study.

**Fig 1:**
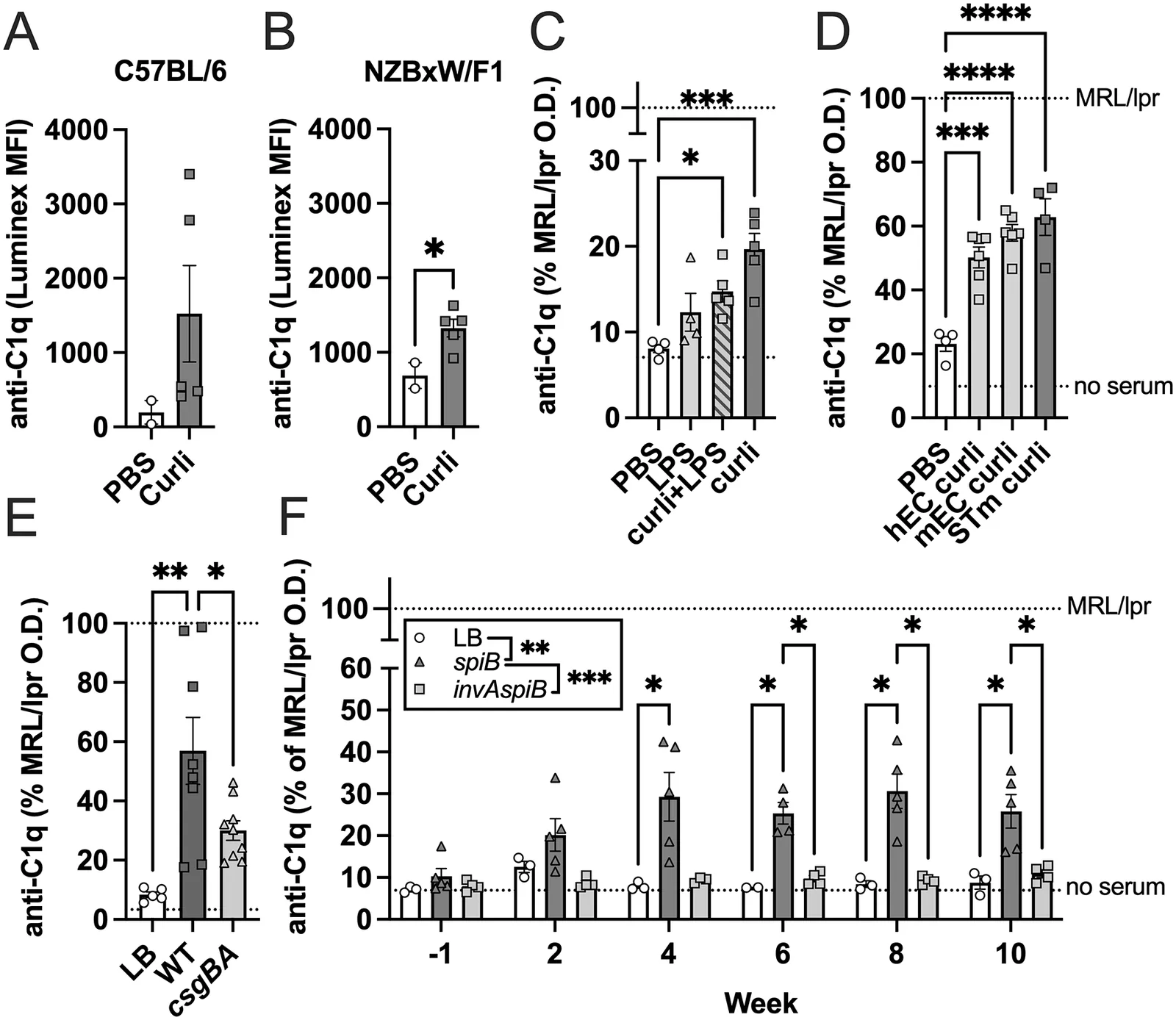
Anti-C1q autoantibodies are induced by curli administration or *Salmonella* Typhimurium infection. (**A-B**) Mouse serum was assayed using a multiplex Luminex platform and antibodies quantified by mean fluorescence intensity (MFI); only anti-C1q antibodies are shown here, see S1 Fig for other antigens. 6-8 week old (**A**) C57BL/6 mice and (**B**) NZBxW/F1 mice were injected intraperitoneally with curli purified from *Salmonella* Typhimurium (STm) biofilms, or sterile PBS for controls, weekly for (**A**) 12 weeks or (**B**) 5 weeks. (**C-F**) Anti-C1q antibodies in serum (diluted 1:50) were detected using ELISA, normalized to aliquots of serum from an untreated aged MRL/lpr mouse as a positive control (1:50, top dotted line) and a negative control without serum (bottom dotted line). (**C**) C57BL/6 mice were injected intraperitoneally with 100 µg curli purified from STm biofilms, 100 µg/kg LPS, 50 µg curli and 50 µg/kg LPS, or PBS for untreated controls, weekly for 3 weeks**.** (**D**) C57BL/6 mice were injected intraperitoneally with 100 µg curli purified from the biofilm of an *E. coli* isolate from the human microbiome (hEC), an *E. coli* isolate from the mouse microbiome (mEC), STm biofilms, or PBS for untreated controls, weekly for 4 weeks**.** (**E**) 129Sv mice were infected intragastrically with 10^7^ CFU of wild-type (WT) STm strain IR715, the isogenic curli-deficient mutant *csgBA*, or LB broth for uninfected controls, in two replicate experiments, and Week 6 serum was assayed. (**F**) C57BL/6 mice were infected intragastrically with 10^7^ CFU of STm *spiB* or *invAspiB* mutant, or LB broth for uninfected controls, and monitored for 10 weeks. Week -1 denotes baseline samples collected from each group one week before the first injection (Week 0). Statistics: (**A-B**) Unpaired t-test, (**C-E**) One-way ANOVA, or (**E**) Mixed-effects analysis, with Tukey’s multiple comparisons, comparisons within the legend indicating overall column factor (*p < 0.05, **p < 0.01, ***p < 0.001, ****p < 0.0001).

To verify the finding of anti-C1q antibodies by the multiplex assay, we tested serum from separate experiments with a validated ELISA specific for anti-C1q antibodies. This assay uses a high-salt buffer to prevent C1q from binding to the Fc region of antibodies in an antigen-independent manner. Anti-C1q levels were compared with those of positive control serum from untreated MRL/lpr mice, another lupus model known to spontaneously produce high levels of anti-C1q autoantibodies [83]. We first examined serum collected from C57BL/6 mice intraperitoneally (i.p.) injected for 3 weeks with purified curli, or a combination of curli and LPS (50% of the individual doses of each component), or PBS as a control. LPS was included because it is a well-characterized and potent bacterial immunostimulatory molecule, allowing us to determine whether anti-C1q autoantibody production reflects a general inflammatory response or is more specifically associated with curli exposure. Mice injected with curli, or with the combination of LPS and curli, developed significantly higher levels of anti-C1q antibodies than mice treated with LPS alone or PBS controls (Fig 1C). The slightly lower response in the curli+LPS group than curli alone is likely due to the decreased dosage of curli. In another experiment, C57BL/6 mice injected for 4 weeks with curli purified from *E. coli* isolates from either the human microbiome (hEC) or the mouse microbiome (mEC) showed comparable anti-C1q antibody levels to those injected with curli purified from STm biofilms; these levels were significantly higher than PBS controls (Fig 1D).

To establish whether anti-C1q antibodies are associated with infection, rather than systemically administered biofilm extracts, we turned to the wild-type 129Sv mouse strain, commonly used in *Salmonella* infection studies because they carry a functional SLC11A1, which allows control of bacterial growth while permitting long-term persistence, in contrast to C57BL/6 mice [84]. We infected wild-type 129Sv mice with wild-type STm or its isogenic *csgBA* mutant deficient in curli production [81]. While bacterial load did not differ significantly between strains (S2 Fig), mice infected with the wild-type STm developed significantly higher levels of anti-C1q antibodies compared to the mice infected with the *csgBA* mutant and uninfected LB controls (Fig 1E). While not statistically significant, the *csgBA* mutant did appear to have more of a response than uninfected mice, which could indicate curli is not the only contributing factor, but curli does seem to be required for a robust response.

To further assess infection-associated responses, we utilized an attenuated strain of STm in C57BL/6 mice. This mutant of STm, *spiB,* lacks functional *Salmonella* type-3 secretion system-2 (encoded within *Salmonella* Pathogenicity Island-2, SPI-2), responsible for intracellular survival in phagocytes [85]. We compared the *spiB* mutant to the isogenic *invAspiB* double mutant, which lacks both type-3 secretion systems (mutations in SPI-1 and SPI-2), impairing both invasion past the gut epithelial barrier and intracellular survival [86]. Mice were similarly colonized with both mutants at 1-week post-infection, but the *invAspiB* mutant began to clear more quickly in some mice than *spiB*, with *invAspiB* showing overall lower colonization over time (S3A Fig). There was low-to-undetectable bacterial load in systemic sites, liver and spleen, in mice infected with either mutant, as expected given the mutations (S3B-C Fig). Oral infection of C57BL/6 mice with *spiB* mutant STm induced significant anti-C1q antibody production by 4 weeks post-infection, compared to uninfected controls and to the *invAspiB* non-invasive mutant (Fig 1F). These findings are consistent with previous results showing that STm invasion is required for anti-dsDNA autoantibody production in response to infection [65]; the anti-C1q response also appears to require invasion.

### Curli increases inflammation in NZBxW/F1 mice

Lupus mouse models, NZBxW/F1 and MRL/lpr mice, develop anti-C1q autoantibodies spontaneously with age [83]. In our experiments, we observed that in NZBxW/F1 mice, at an age prior to spontaneous disease onset in terms of serology or clinical manifestations, levels of anti-C1q antibodies were elevated like in C57BL/6 mice (Fig 1B). Therefore, we next assessed the effects of curli treatment at various times in the NZBxW/F1 model. In our facilities, mice begin developing significant clinical and serological disease manifestations around 22–24 weeks old, with autoantibodies preceding other disease sequelae. Based on prior observations of clinical and serological responses, in additional to reported disease timelines in this model, we have divided mice by age into pre-onset, early-disease, and late-disease groups.

The goal of these experiments was to determine whether curli exposure (i) accelerates onset, (ii) exacerbates autoimmune responses already present, and/or (iii) alters disease manifestations, since anti-C1q autoantibodies are associated with lupus nephritis (LN) in patients. Two of the three age groups were assayed in parallel: the “pre-onset” group began curli or PBS treatments at 8 weeks old and represents genetic predisposition without active disease, while the “early-disease” group began treatments at 22 weeks old and represents mild disease (Fig 2A). The third group, in a separate experiment, was “late-disease”, which started treatments at 28 weeks old and represents severe disease (Fig 2B).

**Fig 2:**
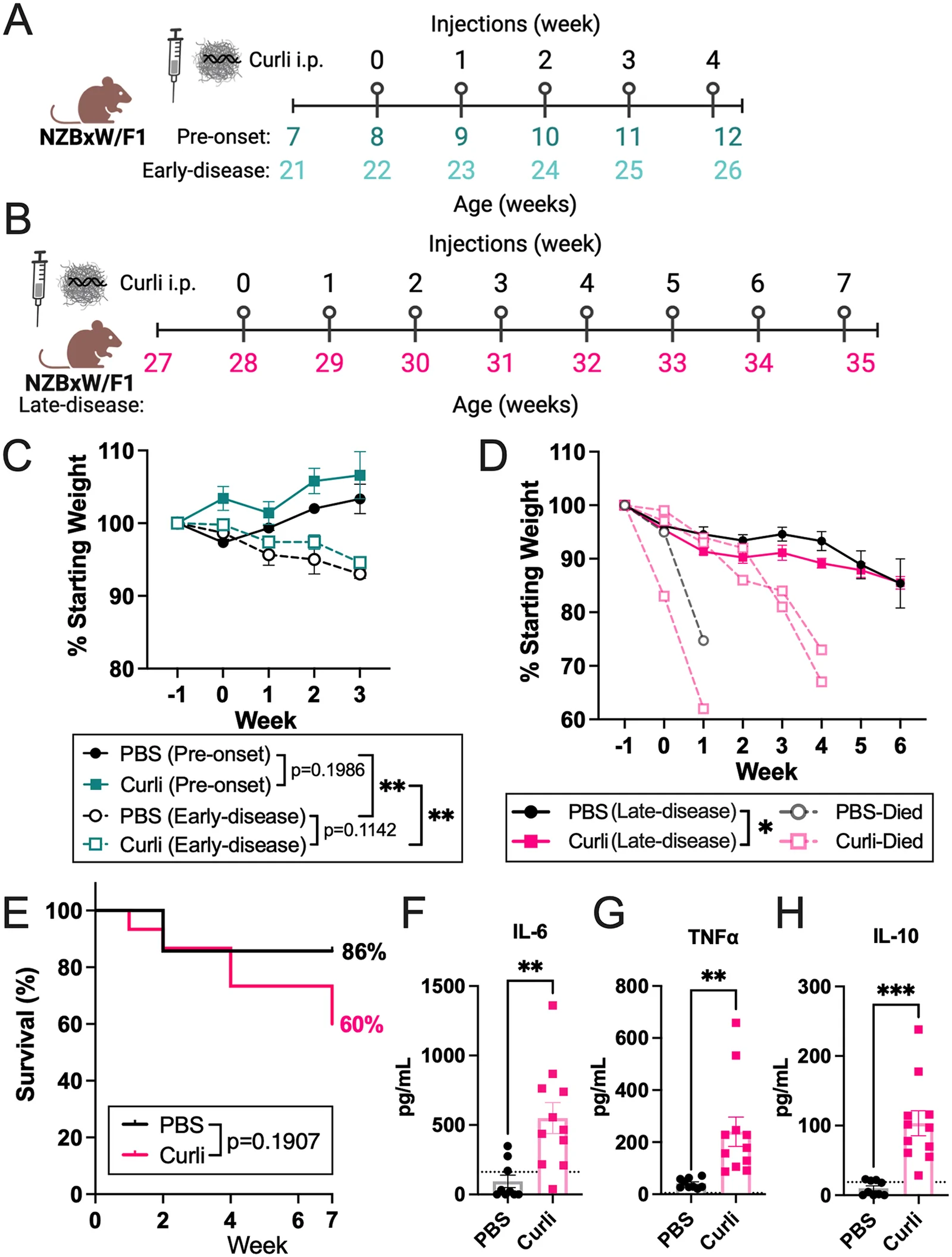
Curli induced cytokine responses in NZBxW/F1 mice. (**A-B**) Experimental scheme: NZBxW/F1 mice starting at age (**A**) 8 weeks (Pre-onset), 22 weeks (Early-disease), or (**B**) 28 weeks (Late-disease) underwent weekly intraperitoneal injections of curli, purified from *Salmonella* Typhimurium biofilms, or PBS for untreated controls. Periodic serum samples were collected 1 week before starting injections and 24 hours after the last injection, weekly for (**A**) 4 weeks or (**B**) 7 weeks. (**C-D**) Weight was tracked over time, normalized to the starting weight 1 week before treatment (**C**, N = 16; **D**, N = 19 Individual late-disease NZBxW/F1 mice that rapidly lost weight and had to be euthanized are shown separately.) (**E**) Late-disease NZBxW/F1 mice were euthanized if 20% weight was lost or predetermined humane endpoints based on pain signs were reached; survival was tracked over time (N = 29, 3 replicate experiments). (**F-H**) Multiplex MSD U-plex ELISA detected (**F**) IL-6, (**G**) TNFα, and (**H**) IL-10 in serum 24 hours after the first curli injection in late-disease NZBxW/F1 mice; dotted line indicates lower detection limit of the assay. Statistics: (**C**) Two-way ANOVA with Tukey’s multiple comparisons, (**D**) Mixed-effects model with Sidak’s multiple comparisons, (**C-D**) comparisons within legend indicating overall column factor, (**E**) Log-rank test, (**F-H**) Unpaired t-test (*p < 0.05, **p < 0.01, ***p < 0.001).

Weight fluctuations are common in patients with lupus and can either result in cachexia, a wasting syndrome, or weight gain by edema associated with kidney dysfunction [87]. Weight in these mice was tracked weekly to monitor for unexpected rapid loss or gain. Pre-onset mice gained weight as they grew, with no abnormal fluctuations or significant differences between curli- and PBS-injected groups (Fig 2C). Early-disease mice began to lose weight as the disease progressed, but there was no observable difference between treatment groups (Fig 2C). Curli-treated late-disease NZBxW/F1 mice weighed less overall, particularly during the early weeks compared to PBS-treated mice, although individual timepoints were not statistically different, and weights converged later in the study (Fig 2D). Some individual mice in the late-disease group—6 of 15 (40%) curli-treated and 2 of 14 (14%) PBS controls—rapidly lost >20% of their starting weight and had to be euthanized before the experimental endpoint; the difference in survival was not statistically significant (Fig 2D-E).

To explore further effects of curli administration on inflammation, acute cytokine responses were examined using an MSD multiplex ELISA panel on serum collected 1 day after the first injection from late-disease NZBxW/F1 mice. Pro-inflammatory IL-6 and TNFα and immunomodulatory IL-10 were all significantly increased after curli treatment (Fig 2F-H), consistent with previous studies in wild-type mice [64,88]. IL-23, which is associated with the Th17 response and strongly implicated in autoimmunity [89], was significantly increased with curli treatment on Day 8, 1 day after the 2^nd^ injection (S4A Fig). IL-1β, IL-27p28/IL-30, IL-15, MIP-1α (CCL3), and MIP-2 (CXCL2) appeared higher after curli treatment but the differences were not statistically significant (S4B-F Fig). These findings indicate that curli can provoke acute cytokine responses despite ongoing autoimmune processes.

### Curli induces anti-C1q autoantibodies in NZBxW/F1 mice

Next, we assessed autoantibody responses during disease progression. As previously demonstrated [64,66,90], NZBxW/F1 mice, at an age prior to onset of clinical disease manifestations, developed accelerated anti-dsDNA antibody responses after curli treatment (Fig 3A). Even in the early-disease group, however, NZBxW/F1 mice appear to already have elevated levels of anti-dsDNA before treatment in both groups, compared to levels observed in pre-onset groups, likely due to the spontaneous autoantibody responses known to occur in this model by this age [91]. These anti-dsDNA levels were increased by week 4 of curli treatment compared to week 0, but the increase was not statistically significant relative to PBS controls (Fig 3B). In late-disease mice, the levels of anti-dsDNA antibodies were already elevated, even before treatment, and curli administration did not demonstrably alter the response (Fig 3C).

**Fig 3:**
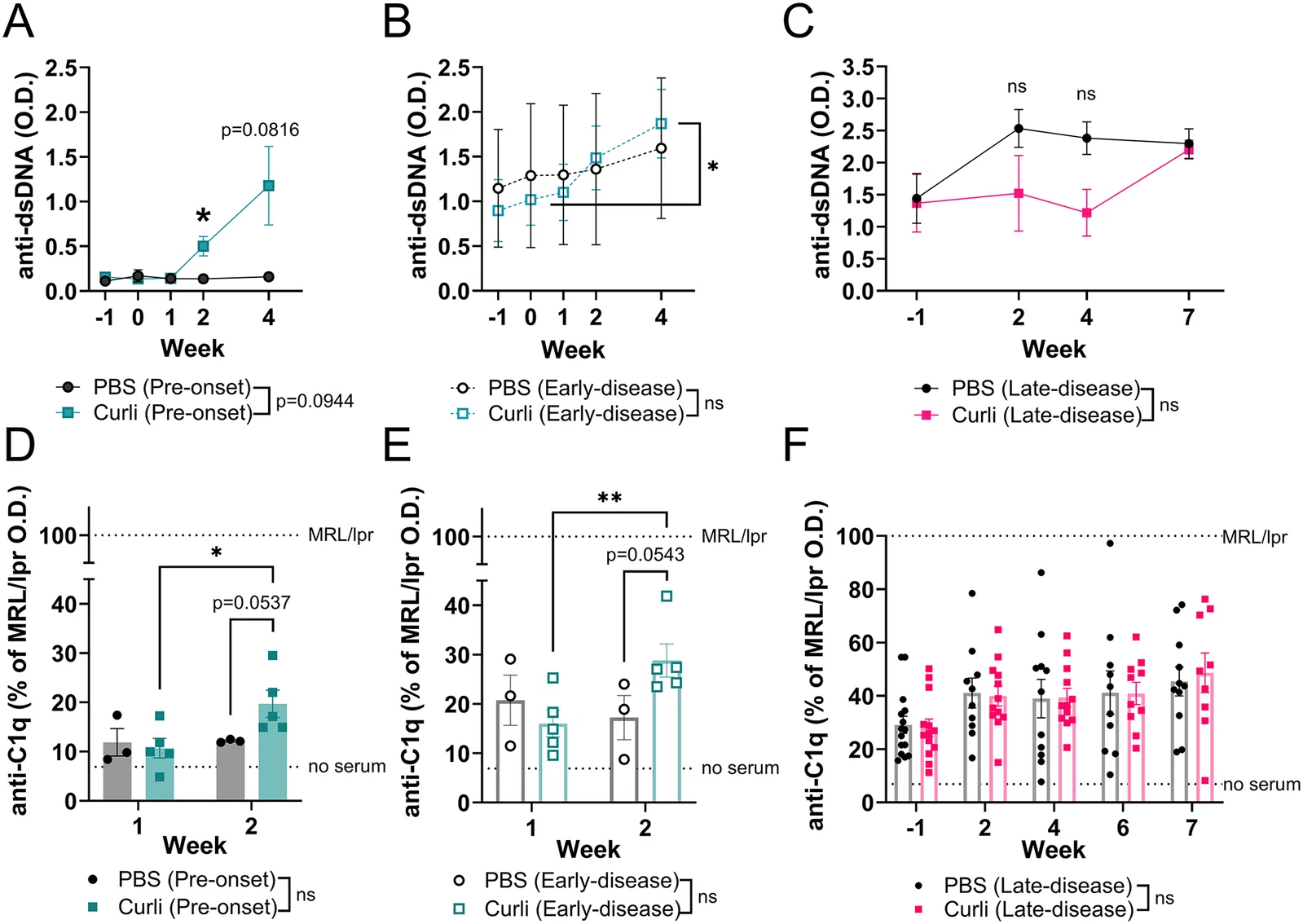
Autoantibody levels are elevated in NZBxW/F1 mice with age and curli treatment. Serum was collected from (**A, D**) pre-onset, (**B, E**) early-disease, and (**C,F**) late-disease NZBxW/F1 mice 24 hours after each weekly injection of curli purified from *Salmonella* Typhimurium biofilms or PBS for untreated controls. (**A-C**) ELISA assays detected anti-dsDNA antibodies in serum diluted (**A, B**) 1:250 or (**C**) 1:500 (**A-B,** N = 8; **C,** N = 15). Week -1 denotes baseline samples collected from each group one week before the first injection (Week 0). (**D-F**) ELISA assays detected anti-C1q antibodies in serum diluted 1:50, normalized to the **O.**D. of positive control MRL/lpr serum, indicated by the top dotted line, and negative controls without serum are indicated by the bottom dotted line (**D-E,** pre-bleed and Week 4 not assayed due to insufficient serum volume for the assay). Statistics: (**A-B, D-E**) Two-way ANOVA, or (**C, F**) Mixed-effects model, with Tukey’s multiple comparisons, comparisons within legends indicating overall column factor, (*p < 0.05, **p < 0.01).

Similarly, anti-C1q antibodies were elevated in both pre-onset and early-disease NZBxW/F1 mice by week 2 of curli treatment, but not in PBS controls, with baseline levels again higher in early-disease than in pre-onset (Fig 3D-E). Consistent with prior studies [83], late-disease mice exhibited elevated baseline anti-C1q levels. Curli treatment did not significantly alter anti-C1q levels overall compared with PBS controls, and there was high variability between individual mice (Fig 3F). We therefore concluded that curli, when encountered prior to the establishment of robust autoantibody responses, can accelerate the development of anti-C1q autoantibodies in NZBxW/F1 mice, whereas differences are no longer readily detectable once antibody levels are already high.

### Curli exacerbates anti-C1q autoantibodies in MRL/lpr mice

Due to the high degree of variation observed in NZBxW/F1 mice, consistent with other studies [92], we tested another mouse model of SLE, MRL/lpr, that presents an accelerated and more severe form of SLE. In this model, mice develop autoantibodies around week 9 and nephritis by approximately week 12 of age, with death occurring around week 17. We injected MRL/lpr mice, beginning at 7 weeks old, with purified curli and ended the experiment at 13 weeks old, making this timeline analogous to the NZBxW/F1 early-disease group to start, but extending further into the disease progression (Fig 4A). All mice survived the course of treatment. All mice, regardless of treatment, continued to gain weight, with the curli-treated group gaining significantly less weight than the PBS controls (S5A Fig).

**Fig 4:**
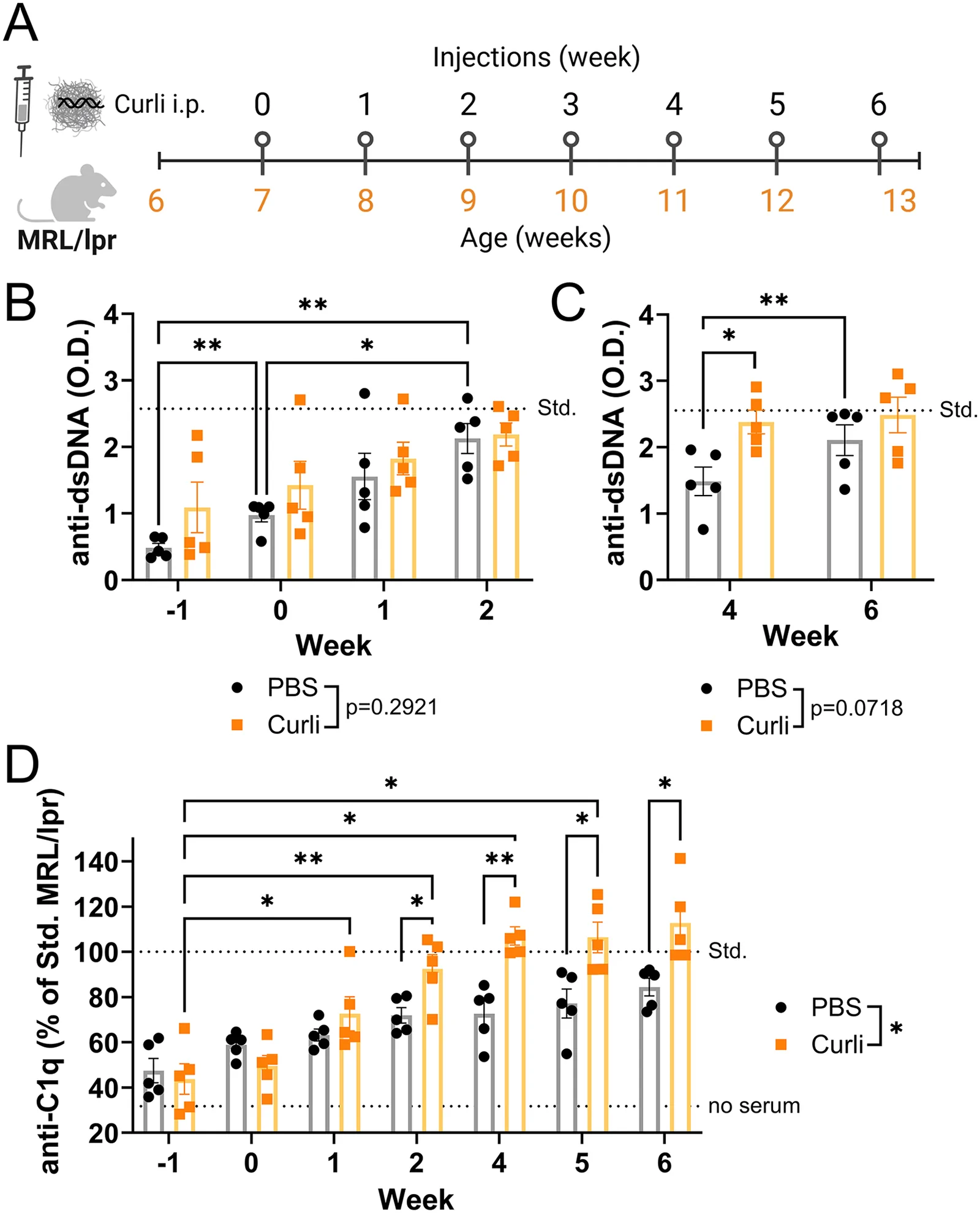
Anti-C1q autoantibody levels are exacerbated in curli-treated MRL/lpr mice. (**A**) Experimental scheme: MRL/lpr mice at age 6 weeks underwent weekly intraperitoneal injections of curli, purified from *Salmonella* Typhimurium biofilms or PBS for untreated controls, and periodic serum samples, taken 24 hours after each injection, for 6 weeks. (**B-C**) ELISA assays detected anti-dsDNA antibodies in serum diluted (**B**) 1:250 or (**C**) 1:500. (**D**) ELISA assay detected anti-C1q antibodies in serum diluted 1:50, normalized to the O.D. of the positive control untreated MRL/lpr serum. Dotted lines indicate relative O.D. of MRL/lpr positive control serum (top line) or negative control with no serum added (bottom line). Week -1 denotes baseline samples collected from each group one week before the first injection (Week 0). Statistics: Two-way ANOVA with Sidak’s multiple comparisons, comparisons within legends indicating overall column factor, (*p < 0.05, **p < 0.01).

Regardless of treatment, all mice had high anti-dsDNA levels by week 2 (Fig 4B). By week 4, mice treated with curli showed significantly increased levels of anti-dsDNA antibodies compared to those of PBS controls, but these responses were similar in both groups by week 6 of treatment (Fig 4C). Strikingly, antibodies against C1q were significantly elevated in curli-treated MRL/lpr mice from week 2–6 of treatment, even higher than those in the positive control, which was collected from untreated MRL/lpr mice (Fig 4D). Overall, these results demonstrate that curli can induce a robust anti-C1q response in MRL/lpr mice, which, in contrast to NZBxW/F1 mice, does not appear to be constrained by a ceiling effect, likely reflecting the defect in immune regulation in the MRL/lpr model.

Since anti-C1q autoantibodies are associated with lupus nephritis in patients [19–24], we assessed kidney function in both NZBxW/F1 and MRL/lpr mice. Urine samples were collected weekly throughout the course of each experiment and proteinuria was measured by sulfosalicylic acid assay. Neither pre-onset nor early-disease NZBxW/F1 mice developed proteinuria, which is expected given their ages (Fig 5A). Among late-disease NZBxW/F1 mice, 57% of curli-treated mice and 39% of PBS controls showed severe proteinuria (>300 mg/dL; Fig 5B-C); this difference was not statistically significant. By the end of the experiment, only one each of curli- and PBS-treated MRL/lpr mice developed severe proteinuria (Fig 5D), an expected result given their age. The Uristix urine dipstick assay, which also measures proteinuria, corroborated the results of the sulfosalicylic acid assay (S5B Fig). Blood urea nitrogen (BUN) was also measured, as another marker of kidney function; again, no significant differences were observed between curli- and PBS-treated MRL/lpr mice (S5C Fig). Since BUN typically spikes late in the progression of nephritis [93], this result is also not unexpected. From these results, we conclude that, at least at the timepoints assayed and in these mouse models, curli exposure does not significantly alter kidney function beyond the established phenotype of each model.

**Fig 5:**
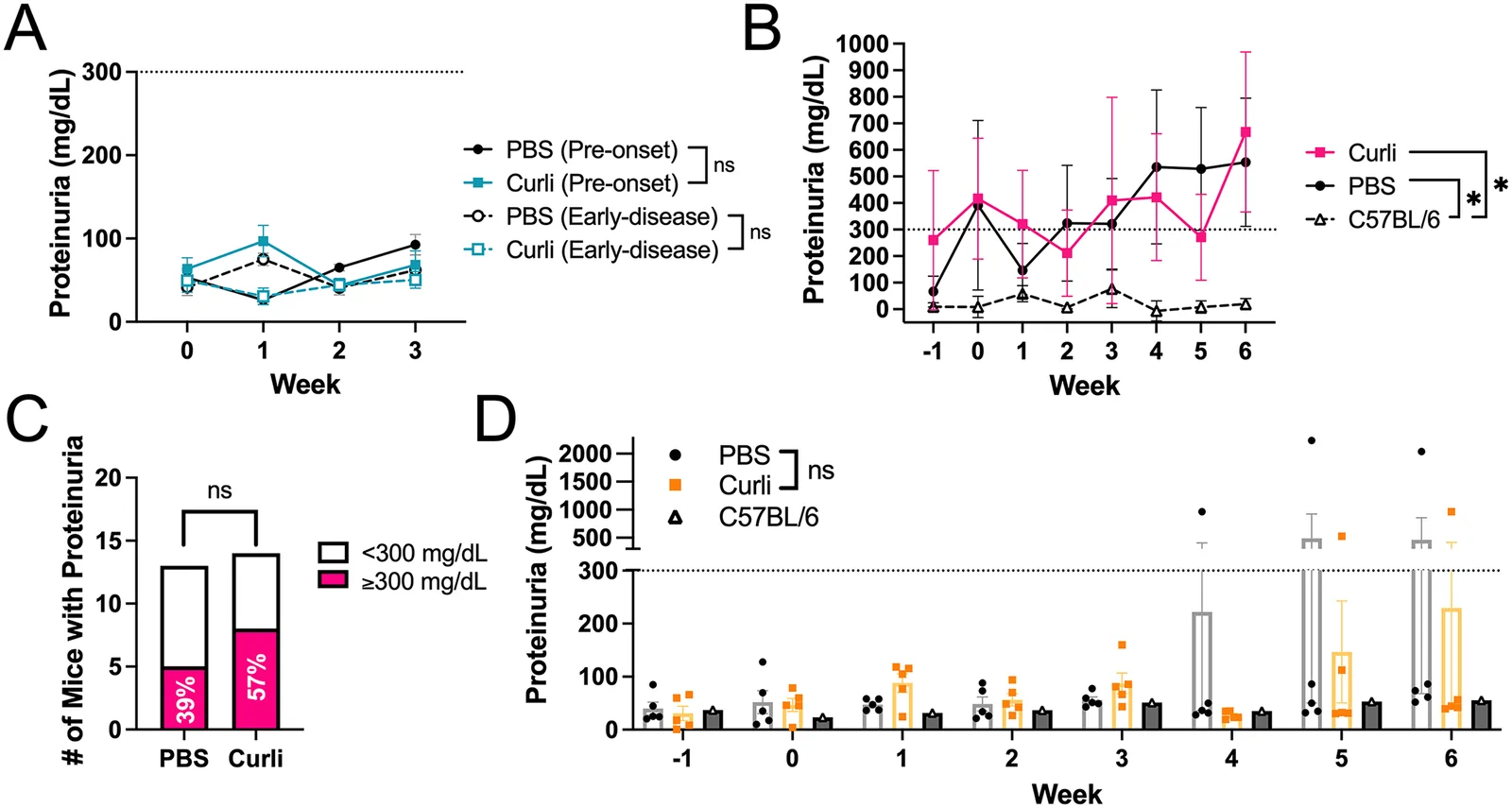
Proteinuria levels in curli-treated NZBxW/F1 and MRL/lpr mice. Urine was collected weekly from (**A**) pre-onset and early-disease NZBxW/F1 (N = 16), (**B-C**) late-disease NZBxW/F1 (N = 27), or (**D**) MRL/lpr mice (N = 10) and assayed for protein levels in urine (proteinuria) by sulfosalicylic acid assay. (**C**) Comparison of the number of late-disease NZBxW/F1 mice that developed proteinuria ≥300 mg/dL over 6 weeks of treatment. Week -1 denotes baseline samples collected from each group one week before the first injection (Week 0). Statistics: (**A-B, D**) Mixed effects analysis with Sidak’s multiple comparisons, comparisons within legend indicating overall column factor, (**C**) Chi-square contingency test (*p < 0.05).

Histopathology scoring of kidneys revealed no apparent pathology in early-disease NZBxW/F1 mice, with varying levels of pathology between individual late-disease NZBxW/F1 mice and no significant differences between PBS and curli-treated groups (S6A Fig). However, it is important to note that only late-disease NZBxW/F1 mice that lived to the experimental endpoint were assessed, which may bias the sample as a few more curli-treated than PBS-treated mice failed to survive through the experiment (Fig 2E). Regarding MRL/lpr mice, only 1 of 5 PBS-treated mice, the same individual with severe proteinuria, developed severe pathology, while 3 of 5 curli-treated MRL/lpr mice developed mild pathology; although this was not statistically significant between groups (S6B Fig). Similar to kidney function assays, curli treatment did not appear to significantly alter kidney pathology at the timepoints examined.

### Treatment with curli increases glomerular C1q deposition in MRL/lpr mice

Another measure of kidney pathology, perhaps more directly related to the role of autoantibodies, is the deposition of immune complexes in glomeruli of the kidneys. Immune complexes in lupus nephritis are typically composed of complement components C3, C1q, and IgG, including the autoantibodies against dsDNA, chromatin, and C1q [25–28,30–32,94]. Kidneys were frozen and sectioned from the same MRL/lpr mice as in Fig 4, which were treated with purified curli or PBS for 6 weeks (Fig 4A). IgG deposition was similar regardless of treatment: both groups had IgG deposits in glomeruli (Fig 6A-C). Similarly, C3 was deposited in high amounts in all but one mouse in each group (Fig 6A-B, D). Notably, the PBS control with no C3 deposition was the same mouse that had developed severe proteinuria 2 weeks earlier (Fig 5D); however, the low C3 deposition in the curli-treated mouse was not the same mouse that had developed proteinuria. Strikingly, significantly higher C1q deposition was consistently observed in the curli-treated MRL/lpr mice than in the PBS controls (Fig 6A-B, E). Average glomerular area was not significantly different between treatments (Fig 6A-B, F), consistent with our histopathological observations (S6B Fig).

**Fig 6:**
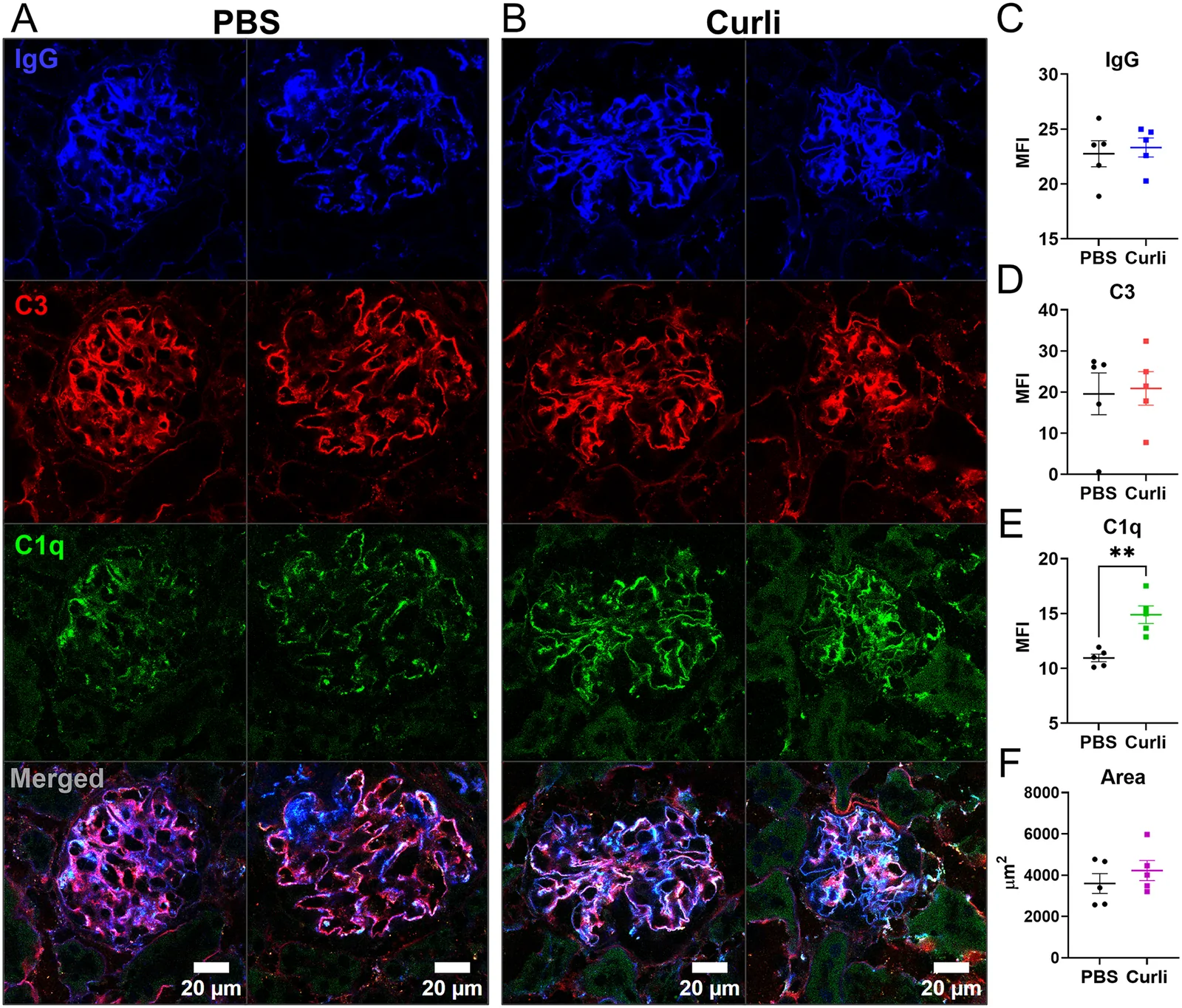
Increased C1q deposition in glomeruli of MRL/lpr mice treated with curli. MRL/lpr mice were intraperitoneally injected weekly with (**A**) PBS or (**B**) curli purified from *Salmonella* Typhimurium for 6 weeks and euthanized at 13 weeks old. Frozen tissue sections were stained with goat anti-mouse IgG-AlexaFluor405 (blue), rat anti-mouse C3-AlexaFluor647 (red), and rat anti-mouse C1q-FITC (green). (**A-B**) Representative images of glomeruli by confocal microscopy (63X objective). Sections shown are from two individual mice per group, scale bars represent 20 µm. (**C-F**) Images were quantified in ImageJ for mean fluorescence intensity (MFI) inside glomeruli of (**C**) IgG, (**D**) C3, (**E**) C1q, and (**F**) area was measured, within at least 15 glomeruli per mouse and averaged. Statistics: (**C-F**) Unpaired t-test (**p < 0.01).

We measured serum C1q levels at the endpoint in these mice to determine whether increased deposition correlates with depleted circulating levels (S6C Fig). Levels were lower overall than those reported in wild-type mice [95], but there was no significant difference between treatment groups. Together, these results suggest a correlation between significantly elevated anti-C1q autoantibodies in curli-treated MRL/lpr mice and elevated C1q deposition in glomeruli of the kidneys, but this did not result in a profound exacerbation of pathology, kidney dysfunction, or depletion of serum C1q levels at the timepoints or mouse models used.

## Discussion

Here, we provide new insight into the induction of autoantibody responses in murine SLE by demonstrating that treatment with curli—isolated from either STm or *E. coli*—or infection with STm, can trigger anti-C1q autoantibody production. These findings extend previous studies that have investigated curli-induced autoimmunity, showing that curli, found in abundance during colonization with biofilms of pathogens (STm) or enteric commensals (*E. coli*), is a bacterial product that, when it escapes from the gut, is seemingly able to break tolerance to both nuclear molecules like DNA and chromatin, and now a soluble serum protein, C1q. Whether the production of anti-C1q autoantibodies is unique to curli or a common feature of many pathogen-associated molecular patterns remains to be seen. Nonetheless. we found no clear evidence of such a response after chronic sub-septic dosing with LPS, another potent immunostimulant (Fig 1C). Consistent with previous findings, invasion through the gut barrier, but not intracellular survival, appears to be necessary for the infection to induce these autoantibodies; the SPI-2 mutant (*spiB*), with deficient intracellular survival, provoked an anti-C1q response, while a non-invasive SPI-1 and SPI-2 mutant (*invAspiB*) showed no anti-C1q response (Fig 1F) [65]. Other studies have also implicated translocation of commensals from the gut in SLE development [96–98]. It is apparent that systemic exposure to such bacterial antigens may be what triggers these autoimmune responses, but the specific immune mechanisms remain to be ascertained.

Relevant to its capacity to break tolerance, the biofilm matrix is decorated with molecules that mimic host molecules. For example, curli is structurally similar to human pathogenic amyloids such as amyloid-β or α-synuclein, or to functional human amyloids such as serum amyloid A [99–101]; this resemblance suggests a mechanism by which bacterial communities can induce autoimmune responses against similarly structured host proteins. It is also possible that, because of curli’s DNA-binding properties [64,75], extracellular DNA can act as a hapten and become immunogenic when bound to highly immunogenic curli fibrils (acting as a carrier), thereby stimulating anti-dsDNA autoantibodies [7,13]. This hapten-carrier model posits that these bacterial products also stimulate TLRs and CD4 + Th cells, leading to an elevated immune response [7,102–104].

Previous studies have shown that *Staphylococcus aureus* peptidoglycan stimulates IgG class switching via TLR2 signaling, leading to anti-dsDNA antibodies [105]. Expansion and translocation of the gut commensal *Mediterraneibacter gnavus* (formerly *Ruminococcus*)—particularly of strains which express a potent TLR2-stimulating lipoglycan—are highly associated with lupus flares and lupus nephritis [96,97,106]. T-bet+ atypical B cells are thought to be major producers of autoantibodies, requiring TLR9 and IFNγ signaling during *P. falciparum* infection [1,107,108]. We have shown that curli/DNA complexes can stimulate TLR2 and TLR9, and induce IFN responses [78–80,90], and that TLR2 and TLR9 are required for an anti-dsDNA response [75]; these findings support the relevance of these same mechanisms in the development of autoantibodies in response to multiple pathogens, especially those producing biofilms containing DNA-binding proteins like curli.

The induction of antibodies to C1q by exposure to curli is somewhat unexpected, given that C1q is a soluble serum protein. However, previous studies demonstrated that C1q binds to the biofilms of curli-expressing *E. coli* but not to those of curli-deficient mutants [109]. In addition, curli has been reported to inhibit complement-mediated killing via the classical pathway, supporting the notion that it interacts directly with complement components, including C1q [109]. These findings suggest that anti-C1q autoantibody production may occur via a hapten–carrier model, in which curli-bound C1q serves as an immunogenic complex that promotes autoantibody production.

To account for the strong anti-C1q response observed in our study, we speculate that its induction may also result from the potent stimulation of TLRs combined with the persistence of amyloid proteins like curli. Curli and other amyloids are extremely resistant to degradation; in fact, human amyloids, such as amyloid-β, can inhibit autophagy-mediated degradation, which is one of the few cellular processes that can break down amyloid aggregates [110]. With impaired degradation and increased inflammatory signaling, it is possible that the immune system has prolonged exposure to immunogenic material (self and foreign) to develop an anti-self response. This potential mechanism is similar to the idea that impaired apoptosis, and impaired clearance of apoptotic bodies, result in immune system responses to cellular debris in SLE [111].

In this study, we found that anti-C1q autoantibodies can be induced in both wild-type and lupus-prone MRL/lpr and NZBxW/F1 mice at different disease stages. Wild-type mice, even when exposed to curli or infected, had lower levels of anti-C1q below the untreated MRL/lpr positive control serum (Fig 1). Untreated NZBxW/F1 mice show increasing levels of anti-C1q antibodies with age [83]; nevertheless, curli administration was able to amplify this response above the anti-C1q levels in untreated mice in both pre-onset and early-disease, but failed to exacerbate already-high levels in late-disease (Fig 3). This pattern suggests a potential ceiling effect in NZBxW/F1 mice once anti-C1q antibodies reach high levels. In contrast, MRL/lpr mice did not exhibit an apparent upper limit, as curli exposure further increased anti-C1q levels beyond those seen in age-matched untreated controls (Fig 4). These differences are consistent with the possibility that regulatory constraints limiting autoantibody escalation may still be operative in NZBxW/F1 mice, whereas such control mechanisms appear impaired or absent in MRL/lpr mice. It is reasonable to suspect that the impaired apoptosis the MRL/lpr strain is known for, due to defective Fas receptor, is responsible.

Here, we found that administration of curli is associated with increased C1q deposition in the glomeruli of MRL/lpr mice. However, this increase did not appear to translate into decreased kidney function as indicated by proteinuria, nor appreciable changes in pathology, at least in these models or at the timepoints assessed. There are two major hypotheses about the relationship between anti-C1q and lupus pathogenesis. The first posits that anti-C1q antibodies sequester C1q, depleting it from circulation and mimicking genetic C1q-deficiency; C1q deficiency is the strongest known genetic risk factor for SLE [112–114]. According to this model, depletion of C1q impairs the clearance of apoptotic bodies, providing increased antigen load, and promoting autoimmune responses against cellular debris [115]. However, there is evidence that anti-C1q antibodies only bind solid-phase C1q, which is somehow conformationally altered (similar to that bound to antibody), but not free soluble C1q as it would appear in circulation [33,116]. In this study, we did not detect a difference in circulating C1q despite the elevated anti-C1q autoantibody levels and elevated C1q deposition in the glomeruli of curli-treated MRL/lpr mice (S6C Fig). Taken together, these findings do not support systemic depletion of circulating C1q as a dominant mechanism. Instead, they are more consistent with a model in which C1q is locally engaged and deposited within inflamed tissues without measurable reduction in serum levels, suggesting that anti-C1q responses may primarily reflect and/or stabilize tissue-bound complement rather than depleting circulating C1q.

The second hypothesis posits that autoantibodies can recruit complement components, forming immune complex deposits, resulting in enhanced complement-mediated tissue damage and localized inflammation at the sites of those complexes [32–35]. A study by Trouw et al. linked anti-C1q autoantibody deposition to glomerular damage only when C1q was also significantly deposited [32]. Our observation of increased C1q glomerular deposition correlating with higher levels of anti-C1q autoantibodies in MRL/lpr mice may lend support to this second hypothesis. However, we did not observe differential C3 deposition between curli-treated mice and controls, suggesting perhaps a shift towards classical pathway activation, but not overall complement fixation.

While we did not observe substantial loss of kidney function in most MRL/lpr mice at the time point studied, relatively early in nephritis pathogenesis, study of a more advanced stage of nephritis could yield a different result. It is worth noting that more curli-treated NZBxW/F1 late-disease mice failed to survive the course of the experiment than PBS-treated controls (Fig 2E). However, this difference was not statistically significant and it is not clear whether fatality resulted from nephritis or other disease manifestations. The difference in the anti-C1q antibody levels in curli-treated NZBxW/F1 mice was also not as extensive as in curli-treated MRL/lpr mice; it is possible that a larger difference in survival would be observed in curli-treated MRL/lpr mice compared to PBS controls later in disease progression. Finally, C1q deficiency in mice does not predispose to SLE-like pathology as strongly as it does in humans [117]. It is possible that there may be additional factors or pathways in which mice and humans differ that contribute to nephritis pathogenesis.

While more work is required to examine the complex relationship between infection and SLE pathogenesis, the results presented herein provide a novel link between curli, an important component of *Salmonella* and *E. coli* biofilms, and nephritis, implicating dysbiosis or infection in driving disease manifestation at various stages of pathogenesis. Since infection in patients with SLE occurs sporadically and frequently, and involves various organisms, this mechanism may help explain the heterogeneity of SLE, and the consequences of dysbiosis and infection on flares and disease outcomes.

## Methods

### Animal models and ethics statement

Mouse strains utilized in this study included C57BL/6 (#000664), Sv129 (#000691), NZBxW/F1 (#100008), and MLR/lpr (#000485) obtained from Jackson Laboratory. Experiments were performed under protocols approved by AAALAC-accredited Temple University Lewis Katz School of Medicine, Institutional Animal Care and Use Committee (IACUC), on file with the NIH Office for the Protection of Research Risks, in accordance with USDA and PHS Policy on Humane Care and Use of Laboratory Animals. C57BL/6 and Sv129 mice were 6–8 weeks old at the beginning of experiments; NZBxW/F1 mice began experiments at 6–8, 21, or 27 weeks old as stated for each experiment; and MRL/lpr mice were 6 weeks old to begin experiments. Age-matched male and female C57BL/6 were pooled as no statistical difference was noted between sexes; only female NZBxW/F1 and MRL/lpr mice were used given the known sex bias in the lupus models [118,119]. Age-matched female Sv129 mice, purchased at the same time from Jackson laboratories, then housed in two different animal facility buildings on the same campus were infected simultaneously, in parallel (both rooms receiving the same treatments), and no significant differences were found between facilities so results were pooled into treatment groups for the purpose of this study (S2 Fig). Mice were monitored for pain and distress behaviors and weight loss; mice were euthanized if >20% weight was lost or other signals of pain and distress were observed as approved by IACUC.

### Bacterial strains and infection

The wild-type STm strain used is *Salmonella enterica* serovar Typhimurium IR715, derived from ATCC 14028 [120] and isogenic *csgBA* curli-deficient mutant [81], *spiB* type 3 secretion system-2 (T3SS-2) mutant [121], *invAspiB* T3SS-1 and T3SS-2 double mutant [121], and *msbB* LPS mutant [122]. The human *E. coli* (hEC) strain was isolated from the microbiome of a patient with *C. difficile* infection by the laboratory of Dr. Joseph Zackular at the Children’s Hospital of Philadelphia and generously shared with us. The mouse *E. coli* (mEC) was isolated from mouse feces from our animal facility, plated on MacConkey Agar to select for enterics, and the presence of *csgA* confirmed by PCR. All strains were grown in Luria-Bertani (LB) broth supplemented with the appropriate antibiotic if resistances were known: either 50 μg/mL nalidixic acid or 100 μg/mL kanamycin, for overnight cultures at 37°C.

For *in vivo* infection, mice were pre-treated by oral gavage with 100 μl of 200 mg/mL streptomycin sulfate (Sigma, S9137-100G), filter-sterilized through a 0.22 μm vacuum filtration system (Millipore, SCGP00525), to ensure a pathogenesis resembling human gastroenteritis [123]. After 24 hours, mice were infected by oral gavage with 10^7^ bacteria, diluted from an overnight culture grown at 37°C in LB broth. Uninfected controls were given sterile LB broth by oral gavage. Inoculum concentration was confirmed by serial dilution, plating, and CFU counting on LB agar supplemented with the appropriate antibiotic for the strain.

Fecal bacterial shedding was monitored weekly in infected mice, and systemic bacterial load was quantified at endpoint, by collecting fresh fecal pellets weekly and a portion of the liver and of the spleen at endpoint in sterile PBS and homogenizing samples. Serial dilutions were plated on LB agar supplemented with either nalidixic acid (for wild-type, *csgBA,* and *spiB*) or kanamycin (for *invAspiB*). CFUs were counted and the total number was calculated and normalized to the mass of the sample. The average detection limit is shown, indicating what a hypothetical 1 colony counted from an undiluted sample of average mass would be (fewer than 1 colony cannot be detected.)

### Curli purification

Curli aggregates were purified from the *msbB* mutant of STm, with LPS incapable of TLR4 activation, as previously described [74,76]. The *msbB* STm was grown in liquid cultures in yeast extract supplemented with casamino acids (YESCA) broth containing 4% DMSO; human and mouse *E. coli* isolates formed more robust biofilms in LB No Salt broth with 4% DMSO, but the rest of the protocol remained consistent with STm. Curli purified following this protocol still contains approximately 200–400 ng of complexed extracellular DNA per milligram of curli, possibly due to curli’s ability to protect DNA from digestion [64,74,75].

### Luminex multiplex autoantibody assay

Serum was collected via cardiac puncture from C57BL/6 or NZBxW/F1 mice treated starting at 6–8 weeks old with PBS or curli weekly for 12 weeks (C57BL/6) or 5 weeks (NZBxW/F1). Serum was assayed using a multiplex assay originally described [124] and refined [125]. The assay used a panel of avidin-coated beads bound to biotinylated antigens including: dsDNA, mouse chromatin, nucleolin, nucleosome, H2A, H2B, RNP/Sm, Sm, La, Ro52, Ro60, C1q, glomerular extract, curli/DNA, *P. copri* K, *Bacteroides*, anti-mouse IgG, P0, P1, P2, Pr3, HMGB1, beta2GP1, cardiolipin, MPO, MDA-BSA, neutravidin, and tetanus toxoid. Serum was assayed at 1:200 dilution, with serum from MRL/lpr mice as a positive control. Average MFI from over 35 beads per analyte per well were quantified.

### ELISA

Blood was collected weekly by tail bleed, starting 1 week before experiments started, and then by cardiac puncture at the endpoint. To separate serum, whole blood was incubated at 37°C for 30 minutes, followed by 10 minutes at 4°C, and then centrifuged for 10–15 minutes at 3400 x g at 4°C. Supernatant was stored at -80°C until use for ELISA testing.

Anti-C1q autoantibodies were measured by ELISA using a modified version of a previously described protocol [83]. MaxiSorp 96-well plates (ThermoFisher) were coated with 50 µl (1 µg/well) of human C1q (Sigma C1740) in PBS (140 mM NaCl, 2.7 mM KCl, 10.1 mM Na_2_HPO_4_, 1.8 mM KH_2_PO_4_, pH 7.4) overnight at 4°C. Plates were washed 3 times with PBS-T (0.075% Tween-20), then blocked with 150 µl of blocking buffer [PBS with 0.3% Tween-20 and 3% bovine serum albumin (BSA)] for at least 1 hour at room temperature with gentle rocking. Plates were washed, then 50 µl of mouse serum diluted 1:50 in high salt buffer (0.3% Tween-20, 3% BSA, in PBS with 1 M NaCl) was incubated overnight at 4°C. After washing, 50 µl of 1:5000 diluted biotin-conjugated goat anti-mouse IgG (JacksonImmuno) in blocking buffer was incubated for 2 hours at room temperature with gentle rocking. Plates were washed, then 50 µl of 1:8000 avidin-alkaline phosphatase (AAP) (Sigma A7294) in blocking buffer was incubated for 2 hours at room temperature with gentle rocking. After washing, 100 µl of N-nitrophenyl phosphate disodium salt hexahydrate (PNPP), 1 tablet dissolved in 20 mL glycine buffer (10 mM glycine, 2.1 mM MgCl_2_, 1 mM ZnCl_2_, pH 10.4), was added and incubated at 37°C until developed (about 15–45 minutes). Absorbance at 405nm was measured using a plate reader (BMG Labtech POLARstar Omega). Aliquots of pooled serum from untreated MRL/lpr mice were used as positive controls, diluted 1:50, the same control used for every assay. Values were normalized to positive control MRL/lpr serum O.D. for clarity, due to batch variation in O.D. and background noise. Statistical comparisons were only made between samples measured on the same batch. Anti-dsDNA autoantibodies were measured as previously described [63,126]. Aliquots of pooled serum from untreated MRL/lpr mice were used as positive controls, diluted 1:500, the same control used for every assay.

Cytokines were measured in serum collected by tail bleed on day 1 (24 hours after the first injection) and day 8 (24 hours after the second injection). Serum cytokines were measured by MSD U-PLEX Custom Biomarker Group 1 (ms) Assay (Meso Scale Discovery, K15069M-1) following the manufacturer’s instructions. Serum C1q was measured in the terminal bleed at Week 6, 1 day after the last injection, using a Mouse C1q ELISA Kit (Abcam, ab291069) following the manufacturer’s instructions.

### Immunofluorescence for immune complexes

Kidneys were collected from MRL/lpr mice at endpoint, 13 weeks old, embedded in Tissue-Tek O.C.T. (Sakura Finetek 4583) and stored frozen at -80°C. Sections were cut at Fox Chase Cancer Center Histopathology Facility. For staining, slides were fixed in pre-chilled acetone at 4°C for 10 minutes in a coplin jar, then washed with PBS-T (pH 7.3 with 0.5% Tween-20) for 10 minutes at room temperature with gentle agitation. Tissue was circled with a Mini PAP Pen (Genemed), then blocked with 10% normal serum in PBS-T for 30 minutes at room temperature. Tissue was washed with PBS-T three times for 5 minutes each at room temperature. Tissue was incubated in antibody cocktails including 1:30 goat anti-mouse IgG conjugated to AlexaFluor 405 (Invitrogen A31553), 1:25 rat anti-mouse C3 (11H9) conjugated to AlexaFluor 647 (Santa Cruz Biotechnology sc-58926), and 1:20 rat anti-mouse C1q (7H8) conjugated to FITC (Santa Cruz Biotechnology sc-58920) diluted in PBS-T with 1% bovine serum albumin for 2 hours at room temperature, covered with a small circle of parafilm and kept in the dark underneath a lid to prevent evaporation and bleaching. Tissue was washed three times with PBS-T for 5 minutes at room temperature, then mounted with VectaShield antifade mounting medium (Vector Laboratories, H-1000) under a coverslip and sealed with clear nail polish.

Kidneys were imaged by confocal microscopy on a TCS SP5 scanning confocal microscope using the LAS AF (Leica) software. Each kidney was scanned starting from the cortex in a straight line longitudinally and the first ≥15 glomeruli were imaged per mouse. Gain and offset settings were unchanged between images, and all replicate slides were stained on the same day and imaged the next day, to eliminate batch effects. Images were processed, and mean fluorescence intensity (MFI) and area were measured on ImageJ2 (Fiji), by manually selecting all glomeruli as regions of interest, and the MFI per channel and area were measured. Background MFI was calculated by selecting 3 negative regions per image and subtracting the average background MFI from the glomerular MFI. Average MFI of glomeruli per mouse was calculated.

### Kidney function analyses

For proteinuria measurements, urine was collected weekly. Mice were held with one hand and the abdomen massaged to provoke urination into a 1.5mL Eppendorf tube. Urine was sampled in this way three times over 24 hours and pooled for analysis. Urine was stored at 4°C for a maximum of 24 hours while the samples were collected before proteinuria assays were performed. Urine was centrifuged gently at 300 x g at 4°C for 3 minutes to pellet cells, if any. Supernatant was diluted 1:10 then 50 µl combined with 200 µl 3% sulfosalicylic acid (Spectrum Chemical Mfg Corp S-596). Bovine serum albumin was used to calculate a standard curve. Samples were incubated for 5 minutes at room temperature and absorbance at 450nm measured on a plate reader (BMG Biotech POLARStar Omega). For some samples, the supernatant was also analyzed with Uristix dipsticks (Siemens 2184) and compared with reference colors for semi-quantitative measurement of proteinuria. Blood urea nitrogen (BUN) was measured on serum samples using the QuantiChrom Urea Assay Kit (BioAssay Systems DIUR-100) following the manufacturer’s instructions.

### Histopathology

Kidneys were collected from NZBxW/F1 and MRL/lpr mice, fixed in 10% formalin for 48 hours, then transferred into 70% ethanol for storage at room temperature until processing. Kidneys were embedded in paraffin, sectioned, and underwent hematoxylin and eosin (H&E) staining at Fox Chase Cancer Center Histopathology Facility. Tissue slides were then blinded and scored by kidney pathologist Dr. Roberto Caricchio at the University of Massachusetts [127] (S1 Table).

### Statistical analysis

Data were analyzed on GraphPad Prism Version 10 and 11 using the appropriate statistical tests as listed in figure legends, including: two-way ANOVA, one-way ANOVA, or mixed-effects analysis with Tukey’s or Sidak’s multiple comparisons; log-rank test for survival; unpaired t-test; or Chi-square contingency test. One significant outlier was identified by ROUT test in the *invAspiB-*infected group (Fig 1C) and was excluded; no other experiments contained statistically significant outliers. Statistical significance was determined as a p-value less than 0.05 after corrections for multiple comparisons when appropriate.

## Supporting information

S1 Fig Multiplex autoantibody assay. Serum was assayed using a multiplex Luminex platform and autoantibodies were quantified by mean fluorescence intensity (MFI). 6–8 week old (**A**) C57BL/6 and (**B**) NZBxW/F1 mice were injected intraperitoneally with curli purified from *Salmonella* Typhimurium (STm) biofilms, or PBS for untreated controls, weekly for (**A**) 12 weeks (N = 7) or (**B**) 5 weeks (N = 7). Statistics: Two-way ANOVA with Tukey’s multiple comparisons (*p < 0.05).
(TIFF)

S2 Fig *Salmonella* curli-deficient mutant infection bacterial load. 129Sv mice were infected intragastrically with 10^7^ CFU of wild-type (WT) STm strain IR715, the isogenic curli-deficient mutant *csgBA*, or LB broth for uninfected controls, and monitored for 6 weeks, in two replicate experiments. Homogenates of (**A**) feces, (**B**) liver, and (**C**) spleen were plated and CFU calculated, normalized to the mass of the sample. The dotted line indicates the average detection limit, the calculated CFU of a single colony from a sample of average mass; below detection limit means no colonies grew from that sample. Statistics: (**A**) Mixed effects model with Sidak’s multiple comparisons, no significant differences were found, (**B-C**) Unpaired t-tests, no significant differences.
(TIFF)

S3 Fig *Salmonella* type-3 secretion system mutants infection bacterial load. C57BL/6 mice were infected intragastrically with 10^7^ CFU of STm *spiB* or *invAspiB* mutant, or LB broth for uninfected controls, and monitored for 10 weeks. Homogenates of (**A**) feces, (**B**) liver, and (**C**) spleen were plated and CFU calculated, normalized to the mass of the sample. The average detection limit indicates the calculated CFU of a single colony from a sample of average mass; below detection limit means no colonies grew from that sample. Statistics: (**A**) Two-way ANOVA with Sidak’s multiple comparisons, comparisons within legend indicating overall column factor, (**B-C**) Unpaired t-tests (*, p < 0.05).
(TIFF)

S4 Fig Curli-induced cytokines in late-disease NZBxW/F1 mice. Multiplex MSD U-plex ELISA detected serum cytokines in Late-disease NZBxW/F1 mice (**A-F**) 1 day after the first i.p. injection of curli or PBS (Day 1) or (**A-B**) 1 day after the second injection (Day 8). Cytokines assayed included (**A**) IL-23, (**B**) IL-1β, (**C**) IL-27p28/IL-30, (**D**) IL-15, (**E**) MIP-1α/CCL3, and (**F**) MIP-2/CXCL2. Statistics: (**A-B**) Two-way ANOVA with Sidak’s multiple comparisons, (**C-F**) Unpaired t-test (*p < 0.05, **p < 0.01).
(TIFF)

S5 Fig Weight and kidney function markers in MRL/lpr mice injected with curli. MRL/lpr mice were injected intraperitoneally with curli purified from *Salmonella* Typhimurium biofilms, or PBS in untreated controls. (**A**) Mice were weighed weekly 6 hours after each injection; weight gain was graphed relative to starting weight 1 week before first curli injection (Week –1) (N = 10). (**B**) Protein levels in urine (proteinuria) were assayed by Uristix dipsticks. (**C**) Blood urea nitrogen (BUN) was measured in serum. Statistics: (**A, C**) Two-way ANOVA (**B**) mixed effects analysis with Sidak‘s multiple comparisons, comparisons within the legends indicate overall column factor (*p < 0.05, **p < 0.01).
(TIFF)

S6 Fig Kidney pathology and serum C1q level. H&E-stained kidney sections were blinded and scored for pathology, including: glomerulonephritis, interstitial nephritis, and vessel pathology in i.p. curli- or PBS-treated (**A**) Late-disease NZBxW/F1 mice and (**B**) MRL/lpr mice. No statistically significant differences were found between treatment groups. Each bar indicates the cumulative scores of individual mice. (**C**) Circulating serum C1q was quantified by ELISA in MRL/lpr mice at endpoint (13 weeks old, Week 6). Statistics: (**A-B**) Two-way ANOVA with Sidak’s multiple comparisons, (**C**) Unpaired t-test.
(TIFF)

S1 Table Kidney histopathology scoring criteria. (DOCX)

S1 File All raw data are available in the supporting information. (XLSX)

## References

[1] Rivera-Correa J, Rodriguez A. Divergent roles of antiself antibodies during infection. Trends Immunol. 2018;39(7):515–22. doi: 10.1016/j.it.2018.04.003 29724608 PMC6386177

[2] Berlin T, Zandman-Goddard G, Blank M, Matthias T, Pfeiffer S, Weis I, et al. Autoantibodies in nonautoimmune individuals during infections. Ann N Y Acad Sci. 2007;1108:584–93. doi: 10.1196/annals.1422.061 17894023

[3] Litwin CM, Binder SR. ANA testing in the presence of acute and chronic infections. J Immunoassay Immunochem. 2016;37(5):439–52. doi: 10.1080/15321819.2016.1174136 27050929

[4] Menzel AEO, Heidelberger M. Cell protein fractions of bovine and avian tubercle bacillus strains and of the timothy-grass bacillus. J Biol Chem. 1938;124(1):301–7. doi: 10.1016/S0021-9258(18)74098-2

[5] Sevag MG, Lackman DB, Smolens J. The isolation of the components of streptococcal nucleoproteins in serologically active form. J Biol Chem. 1938;124(2):425–36. doi: 10.1016/S0021-9258(18)74048-9

[6] Winkenwerder WL, Buell MV, Howard JE. The sensitizing properties of the nucleic acids and their derivatives. Science. 1939;90(2337):356. doi: 10.1126/science.90.2337.356 17843027

[7] Rekvig OP. The anti-DNA antibody: Origin and impact, dogmas and controversies. Nat Rev Rheumatol. 2015;11(9):530–40. doi: 10.1038/nrrheum.2015.69 26034836

[8] Eriksson C, Kokkonen H, Johansson M, Hallmans G, Wadell G, Rantapää-Dahlqvist S. Autoantibodies predate the onset of systemic lupus erythematosus in northern Sweden. Arthritis Res Ther. 2011;13(1):R30. doi: 10.1186/ar3258 21342502 PMC3241374

[9] Arbuckle MR, James JA, Kohlhase KF, Rubertone MV, Dennis GJ, Harley JB. Development of anti-dsDNA autoantibodies prior to clinical diagnosis of systemic lupus erythematosus. Scand J Immunol. 2001;54(1–2):211–9. doi: 10.1046/j.1365-3083.2001.00959.x 11439169

[10] Madaio MP, Hodder S, Schwartz RS, Stollar BD. Responsiveness of autoimmune and normal mice to nucleic acid antigens. J Immunol. 1984;132(2):872–6. doi: 10.4049/jimmunol.132.2.872 6690621

[11] Gilkeson GS, Grudier JP, Karounos DG, Pisetsky DS. Induction of anti-double stranded DNA antibodies in normal mice by immunization with bacterial DNA. J Immunol. 1989;142(5):1482–6. doi: 10.4049/jimmunol.142.5.1482 2645362

[12] Urbonaviciute V, Fürnrohr BG, Meister S, Munoz L, Heyder P, De Marchis F, et al. Induction of inflammatory and immune responses by HMGB1–nucleosome complexes: Implications for the pathogenesis of SLE. J Exp Med. 2008;205(13):3007–18. doi: 10.1084/jem.20081165 19064698 PMC2605236

[13] Qiu CC, Caricchio R, Gallucci S. Triggers of autoimmunity: The role of bacterial infections in the extracellular exposure of lupus nuclear autoantigens. Front Immunol. 2019;10:2608. doi: 10.3389/fimmu.2019.02608 31781110 PMC6857005

[14] Dema B, Charles N. Autoantibodies in SLE: Specificities, isotypes and receptors. Antibodies. 2016;5(1):2. doi: 10.3390/antib5010002 31557984 PMC6698872

[15] Hoover PJ, Costenbader KH. Insights into the epidemiology and management of lupus nephritis from the U.S. Rheumatologist’s perspective. Kidney Int. 2016 Sep;90(3):487–92. doi: 10.1016/j.kint.2016.03.042 27344205 PMC5679458

[16] Almaani S, Meara A, Rovin BH. Update on lupus nephritis. Clin J Am Soc Nephrol. 2017;12(5):825–35. doi: 10.2215/CJN.05780616 27821390 PMC5477208

[17] Teh CL, Phui VE, Ling GR, Ngu L-S, Wan SA, Tan CH-H. Causes and predictors of mortality in biopsy-proven lupus nephritis: The Sarawak experience. Clin Kidney J. 2018;11(1):56–61. doi: 10.1093/ckj/sfx063 29423203 PMC5798016

[18] Giles BM, Boackle SA. Linking complement and anti-dsDNA antibodies in the pathogenesis of systemic lupus erythematosus. Immunol Res. 2013;55(1–3):10–21. doi: 10.1007/s12026-012-8345-z 22941560 PMC4018221

[19] Fava A, Wagner CA, Guthridge CJ, Kheir J, Macwana S, DeJager W, et al. Association of autoantibody concentrations and trajectories with lupus nephritis histologic features and treatment response. Arthritis Rheumatol. 2024;76(11):1611–22. doi: 10.1002/art.42941 38962936 PMC11521769

[20] Akhter E, Burlingame RW, Seaman AL, Magder L, Petri M. Anti-C1q antibodies have higher correlation with flares of lupus nephritis than other serum markers. Lupus. 2011;20(12):1267–74. doi: 10.1177/0961203311411597 21813587

[21] Horváth L, Czirják L, Fekete B, Jakab L, Pozsonyi T, Kalabay L, et al. High levels of antibodies against Clq are associated with disease activity and nephritis but not with other organ manifestations in SLE patients. Clin Exp Rheumatol. 2001;19(6):667–72. 11791638

[22] Marto N, Bertolaccini ML, Calabuig E, Hughes GRV, Khamashta MA. Anti-C1q antibodies in nephritis: Correlation between titres and renal disease activity and positive predictive value in systemic lupus erythematosus. Ann Rheum Dis. 2005;64(3):444–8. doi: 10.1136/ard.2004.024943 15286009 PMC1755385

[23] Siegert CE, Daha MR, Tseng CM, Coremans IE, van Es LA, Breedveld FC. Predictive value of IgG autoantibodies against C1q for nephritis in systemic lupus erythematosus. Ann Rheum Dis. 1993;52(12):851–6. doi: 10.1136/ard.52.12.851 8311534 PMC1005214

[24] Trendelenburg M, Marfurt J, Gerber I, Tyndall A, Schifferli JA. Lack of occurrence of severe lupus nephritis among anti-C1q autoantibody-negative patients. Arthritis Rheum. 1999;42(1):187–8. doi: 10.1002/1529-0131(199901)42:1<187::AID-ANR24>3.0.CO;2-U 9920031

[25] Sterner RM, Hartono SP, Grande JP. The pathogenesis of lupus nephritis. J Clin Cell Immunol. 2014;5(2):205. doi: 10.4172/2155-9899.1000205 25133068 PMC4131745

[26] Weening JJ, D’Agati VD, Schwartz MM, Seshan SV, Alpers CE, Appel GB, et al. The classification of glomerulonephritis in systemic lupus erythematosus revisited. Kidney Int. 2004;65(2):521–30. doi: 10.1111/j.1523-1755.2004.00443.x 14717922

[27] Berden JH, Licht R, van Bruggen MC, Tax WJ. Role of nucleosomes for induction and glomerular binding of autoantibodies in lupus nephritis. Curr Opin Nephrol Hypertens. 1999;8(3):299–306. doi: 10.1097/00041552-199905000-00005 10456260

[28] Kalaaji M, Fenton KA, Mortensen ES, Olsen R, Sturfelt G, Alm P, et al. Glomerular apoptotic nucleosomes are central target structures for nephritogenic antibodies in human SLE nephritis. Kidney Int. 2007;71(7):664–72. doi: 10.1038/sj.ki.5002133 17332738

[29] Kalaaji M, Mortensen E, Jørgensen L, Olsen R, Rekvig OP. Nephritogenic lupus antibodies recognize glomerular basement membrane-associated chromatin fragments released from apoptotic intraglomerular cells. Am J Pathol. 2006;168(6):1779–92. doi: 10.2353/ajpath.2006.051329 16723695 PMC1606630

[30] Grootscholten C, Dieker JWC, McGrath FD, Roos A, Derksen RHWM, van der Vlag J, et al. A prospective study of anti-chromatin and anti-C1q autoantibodies in patients with proliferative lupus nephritis treated with cyclophosphamide pulses or azathioprine/methylprednisolone. Ann Rheum Dis. 2007;66(5):693–6. doi: 10.1136/ard.2006.065425 17135217 PMC1954637

[31] Trendelenburg M, Lopez-Trascasa M, Potlukova E, Moll S, Regenass S, Frémeaux-Bacchi V, et al. High prevalence of anti-C1q antibodies in biopsy-proven active lupus nephritis. Nephrol Dial Transplant. 2006;21(11):3115–21. doi: 10.1093/ndt/gfl436 16877491

[32] Trouw LA, Groeneveld TWL, Seelen MA, Duijs JMGJ, Bajema IM, Prins FA, et al. Anti-C1q autoantibodies deposit in glomeruli but are only pathogenic in combination with glomerular C1q-containing immune complexes. J Clin Invest. 2004;114(5):679–88. doi: 10.1172/JCI21075 15343386 PMC514584

[33] Dijkstra DJ, van de Bovenkamp FS, Abendstein L, Zuijderduijn R, Pool J, Kramer CSM, et al. Human anti-C1q autoantibodies bind specifically to solid-phase C1q and enhance phagocytosis but not complement activation. Proceedings of the National Academy of Sciences. 2023;120(50):e2310666120. doi: 10.1073/pnas.2310666120 PMC10723154 38048459

[34] Holers VM. Anti-C1q autoantibodies amplify pathogenic complement activation in systemic lupus erythematosus. J Clin Invest. 2004;114(5):616–9. doi: 10.1172/JCI22820 15343378 PMC514596

[35] Uwatoko S, Gauthier VJ, Mannik M. Autoantibodies to the collagen-like region of C1Q deposit in glomeruli via C1Q in immune deposits. Clin Immunol Immunopathol. 1991;61(2 Pt 1):268–73. doi: 10.1016/s0090-1229(05)80030-3 1914260

[36] Petri M. Infection in systemic lupus erythematosus. Rheum Dis Clin North Am. 1998;24(2):423–56. doi: 10.1016/s0889-857x(05)70016-8 9606766

[37] Barrera-Vargas A, Gómez-Martín D, Merayo-Chalico J, Ponce-de-León A, Alcocer-Varela J. Risk factors for drug-resistant bloodstream infections in patients with systemic lupus erythematosus. J Rheumatol. 2014;41(7):1311–6. doi: 10.3899/jrheum.131261 24882843

[38] Fessler BJ. Infectious diseases in systemic lupus erythematosus: Risk factors, management and prophylaxis. Best Pract Res Clin Rheumatol. 2002;16(2):281–91. doi: 10.1053/berh.2001.0226 12041954

[39] Staples PJ, Gerding DN, Decker JL, Gordon RS. Incidence of infection in systemic lupus erythematosus. Arthritis Rheum. 1974;17(1):1–10. doi: 10.1002/art.1780170102 4810662

[40] Shahram F, Akbarian M, Davatchi F. Salmonella infection in systemic lupus erythematosus. Lupus. 1993;2(1):55–9. doi: 10.1177/096120339300200110 8485561

[41] Pachucki RJ, Corradetti C, Kohler L, Ghadiali J, Gallo PM, Nicastro L, et al. Persistent bacteriuria and antibodies recognizing curli/eDNA complexes from escherichia coli are linked to flares in systemic lupus erythematosus. Arthritis Rheumatol. 2020;72(11):1872–81. doi: 10.1002/art.41400 32840064 PMC7722165

[42] Tsai Y-C, Hou C-L, Yao T-C, Chen L-C, Jaing T-H, Huang J-L. Risk factors and bacterial profiles of urinary tract infections in patients with systemic lupus erythematosus. Asian Pac J Allergy Immunol. 2007;25(2–3):155–61. 18035803

[43] Battaglia M, Garrett-Sinha LA. Bacterial infections in lupus: Roles in promoting immune activation and in pathogenesis of the disease. J Transl Autoimmun. 2020;4:100078. doi: 10.1016/j.jtauto.2020.100078 33490939 PMC7804979

[44] Gerona JG, Navarra SV. Salmonella infections in patients with systemic lupus erythematosus: A case series. Int J Rheum Dis. 2009;12(4):319–23. doi: 10.1111/j.1756-185X.2009.01440.x 20374369

[45] Costa-Reis P, Nativ S, Isgro J, Rodrigues T, Yildirim-Toruner C, Starr A, et al. Major infections in a cohort of 120 patients with juvenile-onset systemic lupus erythematosus. Clin Immunol. 2013;149(3):442–9. doi: 10.1016/j.clim.2013.08.009 24211846

[46] Lim E, Koh WH, Loh SF, Lam MS, Howe HS. Non-thyphoidal salmonellosis in patients with systemic lupus erythematosus. A study of fifty patients and a review of the literature. Lupus. 2001;10(2):87–92. doi: 10.1191/096120301675973164 11237131

[47] Tsao C-H, Chen C-Y, Ou L-S, Huang J-L. Risk factors of mortality for salmonella infection in systemic lupus erythematosus. J Rheumatol. 2002;29(6):1214–8. 12064838

[48] Abramson S, Kramer SB, Radin A, Holzman R. Salmonella bacteremia in systemic lupus erythematosus. Eight-year experience at a municipal hospital. Arthritis Rheum. 1985;28(1):75–9. doi: 10.1002/art.1780280112 3881103

[49] Chen B, Jia X-M, Xu J-Y, Zhao L-D, Ji J-Y, Wu B-X, et al. An autoimmunogenic and proinflammatory profile defined by the gut microbiota of patients with untreated systemic lupus erythematosus. Arthritis Rheumatol. 2021;73(2):232–43. doi: 10.1002/art.41511 33124780

[50] Zhang L, Qing P, Yang H, Wu Y, Liu Y, Luo Y. Gut microbiome and metabolites in systemic lupus erythematosus: Link, mechanisms and intervention. Front Immunol. 2021;12:686501. doi: 10.3389/fimmu.2021.686501 34335588 PMC8319742

[51] Hevia A, Milani C, López P, Cuervo A, Arboleya S, Duranti S. Intestinal dysbiosis associated with systemic lupus erythematosus. mBio. 2014;5(5):e01548-01514. doi: 10.1128/mBio.01548-14 25271284 PMC4196225

[52] He J, Chan T, Hong X, Zheng F, Zhu C, Yin L, et al. Microbiome and metabolome analyses reveal the disruption of lipid metabolism in systemic lupus erythematosus. Front Immunol. 2020;11:1703. doi: 10.3389/fimmu.2020.01703 32849599 PMC7411142

[53] Wei F, Xu H, Yan C, Rong C, Liu B, Zhou H. Changes of intestinal flora in patients with systemic lupus erythematosus in northeast China. PLoS One. 2019;14(3):e0213063. doi: 10.1371/journal.pone.0213063 30870437 PMC6417672

[54] Shin N-R, Whon TW, Bae J-W. Proteobacteria: Microbial signature of dysbiosis in gut microbiota. Trends Biotechnol. 2015;33(9):496–503. doi: 10.1016/j.tibtech.2015.06.011 26210164

[55] Zhang S-X, Wang J, Chen J-W, Zhang M-X, Zhang Y-F, Hu F-Y, et al. The level of peripheral regulatory T cells is linked to changes in gut commensal microflora in patients with systemic lupus erythematosus. Ann Rheum Dis. 2021;80(11):e177. doi: 10.1136/annrheumdis-2019-216504 31732515

[56] Gui L, Zuo X, Feng J, Wang M, Chen Z, Sun Y, et al. Outgrowth of Escherichia is susceptible to aggravation of systemic lupus erythematosus. Arthritis Res Ther. 2024;26(1):191. doi: 10.1186/s13075-024-03413-7 39511594 PMC11542361

[57] Perkins DJ, Rajaiah R, Tennant SM, Ramachandran G, Higginson EE, Dyson TN, et al. Salmonella Typhimurium Co-Opts the Host Type I IFN System To Restrict Macrophage Innate Immune Transcriptional Responses Selectively. J Immunol. 2015;195(5):2461–71. doi: 10.4049/jimmunol.1500105 26202980 PMC4546913

[58] Winter SE, Thiennimitr P, Winter MG, Butler BP, Huseby DL, Crawford RW, et al. Gut inflammation provides a respiratory electron acceptor for Salmonella. Nature. 2010;467(7314):426–9. doi: 10.1038/nature09415 20864996 PMC2946174

[59] Winter SE, Lopez CA, Bäumler AJ. The dynamics of gut-associated microbial communities during inflammation. EMBO Rep. 2013;14(4):319–27. doi: 10.1038/embor.2013.27 23478337 PMC3615657

[60] Winter SE, Winter MG, Xavier MN, Thiennimitr P, Poon V, Keestra AM, et al. Host-derived nitrate boosts growth of E. coli in the inflamed gut. Science. 2013;339(6120):708–11. doi: 10.1126/science.1232467 PMC4004111 23393266

[61] Rivera-Chávez F, Winter SE, Lopez CA, Xavier MN, Winter MG, Nuccio S-P, et al. Salmonella uses energy taxis to benefit from intestinal inflammation. PLoS Pathog. 2013;9(4):e1003267. doi: 10.1371/journal.ppat.1003267 23637594 PMC3630101

[62] Faber F, Thiennimitr P, Spiga L, Byndloss MX, Litvak Y, Lawhon S, et al. Respiration of microbiota-derived 1,2-propanediol drives Salmonella expansion during colitis. PLoS Pathog. 2017;13(1):e1006129. doi: 10.1371/journal.ppat.1006129 28056091 PMC5215881

[63] Bessho S, Grando KCM, Kyrylchuk K, Miller A, Klein-Szanto AJ, Zhu W, et al. Systemic exposure to bacterial amyloid curli alters the gut mucosal immune response and the microbiome, exacerbating Salmonella-induced arthritis. Gut Microbes. 2023;15(1):2221813. doi: 10.1080/19490976.2023.2221813 37317012 PMC10269392

[64] Gallo PM, Rapsinski GJ, Wilson RP, Oppong GO, Sriram U, Goulian M, et al. Amyloid-DNA composites of bacterial biofilms stimulate autoimmunity. Immunity. 2015;42(6):1171–84. doi: 10.1016/j.immuni.2015.06.002 26084027 PMC4500125

[65] Miller AL, Pasternak JA, Medeiros NJ, Nicastro LK, Tursi SA, Hansen EG, et al. In vivo synthesis of bacterial amyloid curli contributes to joint inflammation during S. Typhimurium infection. PLoS Pathog. 2020;16(7):e1008591. doi: 10.1371/journal.ppat.1008591 32645118 PMC7347093

[66] Nicastro LK, de Anda J, Jain N, Grando KCM, Miller AL, Bessho S, et al. Assembly of ordered DNA-curli fibril complexes during Salmonella biofilm formation correlates with strengths of the type I interferon and autoimmune responses. PLoS Pathog. 2022;18(8):e1010742. doi: 10.1371/journal.ppat.1010742 35972973 PMC9380926

[67] Donlan RM, Costerton JW. Biofilms: Survival mechanisms of clinically relevant microorganisms. Clin Microbiol Rev. 2002;15(2):167–93. doi: 10.1128/CMR.15.2.167-193.2002 11932229 PMC118068

[68] Hathroubi S, Mekni MA, Domenico P, Nguyen D, Jacques M. Biofilms: Microbial shelters against antibiotics. Microb Drug Resist Larchmt N. 2017 Mar;23(2):147–56. doi: 10.1089/mdr.2016.0087 27214143

[69] Costerton JW, Lewandowski Z, Caldwell DE, Korber DR, Lappin-Scott HM. Microbial biofilms. Annual Review of Microbiology. 1995;49:711–45. doi: 10.1146/annurev.mi.49.100195.003431 8561477

[70] Kikuchi T, Mizunoe Y, Takade A, Naito S, Yoshida S. Curli fibers are required for development of biofilm architecture in Escherichia coli K-12 and enhance bacterial adherence to human uroepithelial cells. Microbiol Immunol. 2005;49(9):875–84. doi: 10.1111/j.1348-0421.2005.tb03678.x 16172544

[71] MacKenzie KD, Wang Y, Shivak DJ, Wong CS, Hoffman LJL, Lam S, et al. Bistable expression of CsgD in Salmonella enterica serovar Typhimurium connects virulence to persistence. Infect Immun. 2015;83(6):2312–26. doi: 10.1128/IAI.00137-15 25824832 PMC4432751

[72] Hung C, Zhou Y, Pinkner JS, Dodson KW, Crowley JR, Heuser J. Escherichia coli biofilms have an organized and complex extracellular matrix structure. mBio. 2013;4(5):e00645-00613. doi: 10.1128/mBio.00645-13 24023384 PMC3774191

[73] Hufnagel DA, Tükel C, Chapman MR. Disease to dirt: the biology of microbial amyloids. PLoS Pathog. 2013;9(11):e1003740. doi: 10.1371/journal.ppat.1003740 24278013 PMC3836715

[74] Nicastro LK, Tursi SA, Le LS, Miller AL, Efimov A, Buttaro B. Cytotoxic curli intermediates form during Salmonella biofilm development. J Bacteriol. 2019;201(18). doi: 10.1128/JB.00095-19 31182496 PMC6707925

[75] Tursi SA, Lee EY, Medeiros NJ, Lee MH, Nicastro LK, Buttaro B, et al. Bacterial amyloid curli acts as a carrier for DNA to elicit an autoimmune response via TLR2 and TLR9. PLoS Pathog. 2017;13(4):e1006315. doi: 10.1371/journal.ppat.1006315 28410407 PMC5406031

[76] Grando K, Bessho S, Harrell K, Kyrylchuk K, Pantoja AM, Olubajo S, et al. Bacterial amyloid curli activates the host unfolded protein response via IRE1α in the presence of HLA-B27. Gut Microbes. 2024;16(1):2392877. doi: 10.1080/19490976.2024.2392877 39189642 PMC11352795

[77] Cruz-Cruz A, Schreeg ME, Gunn JS. A temporary cholesterol-rich diet and bacterial extracellular matrix factors favor Salmonella spp. biofilm formation in the cecum. mBio. 2025;16(1):e0324224. doi: 10.1128/mbio.03242-24 39636114 PMC11708031

[78] Tükel C, Raffatellu M, Humphries AD, Wilson RP, Andrews-Polymenis HL, Gull T, et al. CsgA is a pathogen-associated molecular pattern of Salmonella enterica serotype Typhimurium that is recognized by Toll-like receptor 2. Mol Microbiol. 2005;58(1):289–304. doi: 10.1111/j.1365-2958.2005.04825.x 16164566

[79] Tükel C, Nishimori JH, Wilson RP, Winter MG, Keestra AM, van Putten JPM, et al. Toll-like receptors 1 and 2 cooperatively mediate immune responses to curli, a common amyloid from enterobacterial biofilms. Cell Microbiol. 2010;12(10):1495–505. doi: 10.1111/j.1462-5822.2010.01485.x 20497180 PMC3869100

[80] Rapsinski GJ, Wynosky-Dolfi MA, Oppong GO, Tursi SA, Wilson RP, Brodsky IE, et al. Toll-like receptor 2 and NLRP3 cooperate to recognize a functional bacterial amyloid, curli. Infect Immun. 2015;83(2):693–701. doi: 10.1128/IAI.02370-14 25422268 PMC4294241

[81] Nishimori JH, Newman TN, Oppong GO, Rapsinski GJ, Yen J-H, Biesecker SG, et al. Microbial amyloids induce interleukin 17A (IL-17A) and IL-22 responses via Toll-like receptor 2 activation in the intestinal mucosa. Infect Immun. 2012;80(12):4398–408. doi: 10.1128/IAI.00911-12 23027540 PMC3497426

[82] Verstraelen P, Van Remoortel S, De Loose N, Verboven R, Garcia-Diaz Barriga G, Christmann A, et al. Serum Amyloid A3 fuels a feed-forward inflammatory response to the bacterial amyloid curli in the enteric nervous system. Cell Mol Gastroenterol Hepatol. 2024;18(1):89–104. doi: 10.1016/j.jcmgh.2024.03.013 38556049 PMC11127031

[83] Hogarth MB, Norsworthy PJ, Allen PJ, Trinder PK, Loos M, Morley BJ, et al. Autoantibodies to the collagenous region of C1q occur in three strains of lupus-prone mice. Clin Exp Immunol. 1996;104(2):241–6. doi: 10.1046/j.1365-2249.1996.19725.x 8625515 PMC2200417

[84] Monack DM, Bouley DM, Falkow S. Salmonella typhimurium persists within macrophages in the mesenteric lymph nodes of chronically infected Nramp1+/+ mice and can be reactivated by IFNgamma neutralization. J Exp Med. 2004;199(2):231–41. doi: 10.1084/jem.20031319 14734525 PMC2211772

[85] Ochman H, Soncini FC, Solomon F, Groisman EA. Identification of a pathogenicity island required for Salmonella survival in host cells. Proc Natl Acad Sci U S A. 1996;93(15):7800–4. doi: 10.1073/pnas.93.15.7800 8755556 PMC38828

[86] Galán JE, Curtiss R. Cloning and molecular characterization of genes whose products allow Salmonella typhimurium to penetrate tissue culture cells. Proc Natl Acad Sci U S A. 1989;86(16):6383–7. doi: 10.1073/pnas.86.16.6383 2548211 PMC297844

[87] Stojan G, Li J, Wittmaack A, Petri M. Cachexia in systemic lupus erythematosus: risk factors and relation to disease activity and damage. Arthritis Care Res (Hoboken). 2021;73(11):1577–82. doi: 10.1002/acr.24395 32741060 PMC7855238

[88] Oppong GO, Rapsinski GJ, Tursi SA, Biesecker SG, Klein-Szanto AJP, Goulian M, et al. Biofilm-associated bacterial amyloids dampen inflammation in the gut: Oral treatment with curli fibres reduces the severity of hapten-induced colitis in mice. npj Biofilms Microbiomes. 2015;1(1). doi: 10.1038/npjbiofilms.2015.19 PMC4739805 26855788

[89] Lohr J, Knoechel B, Wang JJ, Villarino AV, Abbas AK. Role of IL-17 and regulatory T lymphocytes in a systemic autoimmune disease. J Exp Med. 2006;203(13):2785–91. doi: 10.1084/jem.20061341 PMC2118184 17130300

[90] Gallucci S, Gallo P, Rapsinski G, Oppong G, Sriram U, Wilson R, et al. Bacterial amyloids promote type I interferon production and accelerate autoimmunity (BA6P.124). J Immunol. 2015;194(1_Supplement):114.5. doi: 10.4049/jimmunol.194.Supp.114.5

[91] Wofsy D, Seaman WE. Successful treatment of autoimmunity in NZB/NZW F1 mice with monoclonal antibody to L3T4. J Exp Med. 1985;161(2):378–91. doi: 10.1084/jem.161.2.378 3919141 PMC2187572

[92] Böhme R, Daniel C, Ferrazzi F, Angeloni M, Ekici AB, Winkler TH, et al. Cardiovascular changes in the NZB/W F1 mouse model of lupus nephritis. Front Cardiovasc Med. 2023;10:1182193. doi: 10.3389/fcvm.2023.1182193 37554366 PMC10405627

[93] Dent EL, Taylor EB, Sasser JM, Ryan MJ. Temporal hemodynamic changes in a female mouse model of systemic lupus erythematosus. Am J Physiol Renal Physiol. 2020;318(5):F1074–85. doi: 10.1152/ajprenal.00598.2019 32150445 PMC7294344

[94] Rekvig OP, Nossent JC. Anti-double-stranded DNA antibodies, nucleosomes, and systemic lupus erythematosus: A time for new paradigms?. Arthritis Rheum. 2003;48(2):300–12. doi: 10.1002/art.10739 12571837

[95] Byrne RAJ, Torvell M, Daskoulidou N, Fathalla D, Kokkali E, Carpanini SM, et al. Novel Monoclonal Antibodies Against Mouse C1q: Characterisation and Development of a Quantitative ELISA for Mouse C1q. Mol Neurobiol. 2021;58(9):4323–36. doi: 10.1007/s12035-021-02419-5 34002346 PMC8487419

[96] Azzouz D, Omarbekova A, Heguy A, Schwudke D, Gisch N, Rovin BH, et al. Lupus nephritis is linked to disease-activity associated expansions and immunity to a gut commensal. Ann Rheum Dis. 2019;78(7):947–56. doi: 10.1136/annrheumdis-2018-214856 30782585 PMC6585303

[97] Azzouz DF, Chen Z, Izmirly PM, Chen LA, Li Z, Zhang C, et al. Longitudinal gut microbiome analyses and blooms of pathogenic strains during lupus disease flares. Ann Rheum Dis. 2023;82(10):1315–27. doi: 10.1136/ard-2023-223929 37365013 PMC10511964

[98] Manfredo Vieira S, Hiltensperger M, Kumar V, Zegarra-Ruiz D, Dehner C, Khan N, et al. Translocation of a gut pathobiont drives autoimmunity in mice and humans. Science. 2018;359(6380):1156–61. doi: 10.1126/science.aar7201 29590047 PMC5959731

[99] Dueholm MS, Nielsen SB, Hein KL, Nissen P, Chapman M, Christiansen G, et al. Fibrillation of the major curli subunit CsgA under a wide range of conditions implies a robust design of aggregation. Biochemistry. 2011;50(39):8281–90. doi: 10.1021/bi200967c 21877724 PMC3724407

[100] Sleutel M, Van den Broeck I, Van Gerven N, Feuillie C, Jonckheere W, Valotteau C, et al. Nucleation and growth of a bacterial functional amyloid at single-fiber resolution. Nat Chem Biol. 2017;13(8):902–8. doi: 10.1038/nchembio.2413 28628096 PMC5580806

[101] Chapman MR, Robinson LS, Pinkner JS, Roth R, Heuser J, Hammar M. Role of Escherichia coli curli operons in directing amyloid fiber formation. Science. 2002;295(5556):851–5. doi: 10.1126/science.1067484 11823641 PMC2838482

[102] Van Ghelue M, Moens U, Bendiksen S, Rekvig OP. Autoimmunity to nucleosomes related to viral infection: A focus on hapten-carrier complex formation. J Autoimmun. 2003;20(2):171–82. doi: 10.1016/s0896-8411(02)00110-5 12657530

[103] Rock KL, Benacerraf B, Abbas AK. Antigen presentation by hapten-specific B lymphocytes. I. Role of surface immunoglobulin receptors. J Exp Med. 1984;160(4):1102–13. doi: 10.1084/jem.160.4.1102 6207262 PMC2187484

[104] Desai DD, Krishnan MR, Swindle JT, Marion TN. Antigen-specific induction of antibodies against native mammalian DNA in nonautoimmune mice. J Immunol. 1993;151(3):1614–26. doi: 10.4049/jimmunol.151.3.1614 8393048

[105] Ning W, Cheng D, Howe PH, Bian C, Kamen DL, Luo Z, et al. Staphylococcus aureus peptidoglycan (PGN) induces pathogenic autoantibody production via autoreactive B cell receptor clonal selection, implications in systemic lupus erythematosus. J Autoimmun. 2022;131:102860. doi: 10.1016/j.jaut.2022.102860 35810689 PMC9397544

[106] Amarnani A, Rivera CF, Cornwell M, Weinstein T, Gottesman SRS, Loomis C. Pathogenic strains of a gut commensal drive systemic platelet activation and thromboinflammation in lupus nephritis. bioRxiv. 2025. doi: 10.1101/2025.06.20.641288

[107] Rubtsova K, Rubtsov AV, Thurman JM, Mennona JM, Kappler JW, Marrack P. B cells expressing the transcription factor T-bet drive lupus-like autoimmunity. J Clin Invest. 2017;127(4):1392–404. doi: 10.1172/JCI91250 28240602 PMC5373868

[108] Rivera-Correa J, Guthmiller JJ, Vijay R, Fernandez-Arias C, Pardo-Ruge MA, Gonzalez S. Plasmodium DNA-mediated TLR9 activation of T-bet B cells contributes to autoimmune anaemia during malaria. Nature Communications. 2017;8(1):1282. doi: 10.1038/s41467-017-01476-6 29101363 PMC5670202

[109] Biesecker SG, Nicastro LK, Wilson RP, Tükel Ç. The functional amyloid curli protects Escherichia coli against complement-mediated bactericidal activity. Biomolecules. 2018;8(1):5. doi: 10.3390/biom8010005 29364839 PMC5871974

[110] Yuan M, Wang Y, Huang Z, Jing F, Qiao P, Zou Q, et al. Impaired autophagy in amyloid-beta pathology: A traditional review of recent Alzheimer’s research. J Biomed Res. 2022;37(1):30–46. doi: 10.7555/JBR.36.20220145 36642915 PMC9898044

[111] Gaipl US, Voll RE, Sheriff A, Franz S, Kalden JR, Herrmann M. Impaired clearance of dying cells in systemic lupus erythematosus. Autoimmun Rev. 2005;4(4):189–94. doi: 10.1016/j.autrev.2004.10.007 15893710

[112] Truedsson L, Bengtsson AA, Sturfelt G. Complement deficiencies and systemic lupus erythematosus. Autoimmunity. 2007;40(8):560–6. doi: 10.1080/08916930701510673 18075790

[113] Li NL, Birmingham DJ, Rovin BH. Expanding the role of complement therapies: The case for lupus nephritis. J Clin Med. 2021;10(4):626. doi: 10.3390/jcm10040626 33562189 PMC7915321

[114] van Schaarenburg RA, Schejbel L, Truedsson L, Topaloglu R, Al-Mayouf SM, Riordan A, et al. Marked variability in clinical presentation and outcome of patients with C1q immunodeficiency. J Autoimmun. 2015;62:39–44. doi: 10.1016/j.jaut.2015.06.002 26119135

[115] Botto M, Walport MJ. C1q, autoimmunity and apoptosis. Immunobiology. 2002;205(4–5):395–406. doi: 10.1078/0171-2985-00141 12396002

[116] Bigler C, Schaller M, Perahud I, Osthoff M, Trendelenburg M. Autoantibodies against complement C1q specifically target C1q bound on early apoptotic cells1. J Immunol. 2009;183(5):3512–21. doi: 10.4049/jimmunol.0803573 19648280

[117] Mitchell DA, Pickering MC, Warren J, Fossati-Jimack L, Cortes-Hernandez J, Cook HT, et al. C1q deficiency and autoimmunity: The effects of genetic background on disease expression. J Immunol. 2002;168(5):2538–43. doi: 10.4049/jimmunol.168.5.2538 11859149

[118] Li W, Titov AA, Morel L. An update on lupus animal models. Curr Opin Rheumatol. 2017;29(5):434–41. doi: 10.1097/BOR.0000000000000412 28537986 PMC5815391

[119] Gulinello M, Putterman C. The MRL/lpr mouse strain as a model for neuropsychiatric systemic lupus erythematosus. J Biomed Biotechnol. 2011;2011:207504. doi: 10.1155/2011/207504 21331367 PMC3038428

[120] Stojiljkovic I, Bäumler AJ, Heffron F. Ethanolamine utilization in Salmonella typhimurium: Nucleotide sequence, protein expression, and mutational analysis of the cchA cchB eutE eutJ eutG eutH gene cluster. J Bacteriol. 1995;177(5):1357–66. doi: 10.1128/jb.177.5.1357-1366.1995 7868611 PMC176743

[121] Raffatellu M, George MD, Akiyama Y, Hornsby MJ, Nuccio S-P, Paixao TA, et al. Lipocalin-2 resistance confers an advantage to Salmonella enterica serotype Typhimurium for growth and survival in the inflamed intestine. Cell Host Microbe. 2009;5(5):476–86. doi: 10.1016/j.chom.2009.03.011 19454351 PMC2768556

[122] Raffatellu M, Chessa D, Wilson RP, Dusold R, Rubino S, Bäumler AJ. The Vi capsular antigen of Salmonella enterica serotype Typhi reduces Toll-like receptor-dependent interleukin-8 expression in the intestinal mucosa. Infect Immun. 2005;73(6):3367–74. doi: 10.1128/IAI.73.6.3367-3374.2005 15908363 PMC1111811

[123] Barthel M, Hapfelmeier S, Quintanilla-Martínez L, Kremer M, Rohde M, Hogardt M, et al. Pretreatment of mice with streptomycin provides a Salmonella enterica serovar Typhimurium colitis model that allows analysis of both pathogen and host. Infect Immun. 2003;71(5):2839–58. doi: 10.1128/IAI.71.5.2839-2858.2003 12704158 PMC153285

[124] Tatsuta T, Hosono M, Takahashi K, Omoto T, Kariya Y, Sugawara S, et al. Sialic acid-binding lectin (leczyme) induces apoptosis to malignant mesothelioma and exerts synergistic antitumor effects with TRAIL. Int J Oncol. 2014;44(2):377–84. doi: 10.3892/ijo.2013.2192 24297392 PMC3898873

[125] Pelzek AJ, Grönwall C, Rosenthal P, Greenberg JD, McGeachy M, Moreland L, et al. Persistence of Disease-Associated Anti-Citrullinated Protein Antibody-Expressing Memory B Cells in Rheumatoid Arthritis in Clinical Remission. Arthritis Rheumatol. 2017;69(6):1176–86. doi: 10.1002/art.40053 28118534 PMC5569882

[126] Sriram U, Varghese L, Bennett HL, Jog NR, Shivers DK, Ning Y, et al. Myeloid dendritic cells from B6.NZM Sle1/Sle2/Sle3 lupus-prone mice express an IFN signature that precedes disease onset. J Immunol. 2012;189(1):80–91. doi: 10.4049/jimmunol.1101686 22661089 PMC3381850

[127] Fike AJ, Bricker KN, Gonzalez MV, Maharjan A, Bui T, Nuon K, et al. IRF7 controls spontaneous autoimmune germinal center and plasma cell checkpoints. J Exp Med. 2025;222(7):e20231882. doi: 10.1084/jem.20231882 PMC12121627 40439584

