# Supplementary material for: Salmonella infection and biofilms drive anti-C1q and anti-dsDNA autoantibodies in systemic autoimmunity
Source: PLoS Pathog. 2026 Oct 1;22(10):e1014666. doi: 10.1371/journal.ppat.1014666 (PMC13652334; doi:10.1371/journal.ppat.1014666)
Supplement: Supplementary file 1 [file ppat.1014666.s007.docx]

**S1 Table.**

| Numerical Score | Pathology Score | Glomerulonephritis | Interstitial Nephritis | Vessels |
| --- | --- | --- | --- | --- |
| 0  0.5 | 0-1  0-1+ | Focal, mild, or early proliferative | Occasional, focal, or small pockets of MNC (10-15 cells) | Occasional perivascular infiltrate |
| 1  1.5 | 1-2  1-2+ | Moderate or definite proliferative; increased matrix | Focal infiltrates  (15-30 cells) | Several foci of perivascular infiltrate; no necrosis |
| 2  2.5 | 2-3  2-3+ | Diffuse and focal or diffuse proliferative | Multifocal extensive infiltrates; with necrosis | Multifocal perivascular; more extensive +/- necrosis (3+) |
| 3  3.5 | 3-4  3-4+ | Severe diffuse proliferative, with crescents/sclerosis | Severe disease with extensive necrosis | Multifocal or diffuse; extensive with necrosis |
